# Regulatory T Cells Restrain Interleukin-2- and Blimp-1-Dependent Acquisition of Cytotoxic Function by CD4^+^ T Cells

**DOI:** 10.1016/j.immuni.2019.12.007

**Published:** 2020-01-14

**Authors:** Anna Śledzińska, Maria Vila de Mucha, Katharina Bergerhoff, Alastair Hotblack, Dafne Franz Demane, Ehsan Ghorani, Ayse U. Akarca, Maria A.V. Marzolini, Isabelle Solomon, Frederick Arce Vargas, Martin Pule, Masahiro Ono, Benedict Seddon, George Kassiotis, Charlotte E. Ariyan, Thomas Korn, Teresa Marafioti, Graham M. Lord, Hans Stauss, Richard G. Jenner, Karl S. Peggs, Sergio A. Quezada

**Affiliations:** 1 Cancer Immunology Unit, UCL Cancer Institute, University College London, London WC1E 6DD, UK; 2 Research Department of Haematology, University College London, Cancer Institute, London WC1E 6DD, UK; 3 Regulatory Genomics Research Group, UCL Cancer Institute, University College London, London WC1E 6DD, UK; 4 Department of Cellular Pathology, University College London Hospital, London NW1 2BU, UK; 5 Faculty of Natural Sciences, Department of Life Sciences, Imperial College London, London SW7 2BB, UK; 6 Institute of Immunity and Transplantation, Department of Immunology, Royal Free Hospital, London NW3 2PF, UK; 7 Retroviral Immunology Laboratory, The Francis Crick Institute, 1 Midland Road, London NW1 1AT, UK; 8 Memorial Sloan Kettering Center, 1275 York Avenue, New York, NY 10065, USA; 9 Department of Experimental Neuroimmunology, Klinikum rechts der Isar, Technical University of Munich, 81675 Munich, Germany; 10 Faculty of Biology, Medicine and Health, University of Manchester, 46 Grafton Street, Manchester M13 9NT, UK

**Keywords:** cytotoxic CD4^+^ T cells, regulatory T cells, Treg depletion, anti-CTLA-4, Blimp-1, T-bet, IL-2, CD4-mediated anti-tumor response

## Abstract

In addition to helper and regulatory potential, CD4^+^ T cells also acquire cytotoxic activity marked by granzyme B (GzmB) expression and the ability to promote rejection of established tumors. Here, we examined the molecular and cellular mechanisms underpinning the differentiation of cytotoxic CD4^+^ T cells following immunotherapy. CD4^+^ transfer into lymphodepleted animals or regulatory T (Treg) cell depletion promoted GzmB expression by tumor-infiltrating CD4^+^, and this was prevented by interleukin-2 (IL-2) neutralization. Transcriptional analysis revealed a polyfunctional helper and cytotoxic phenotype characterized by the expression of the transcription factors T-bet and Blimp-1. While T-bet ablation restricted interferon-γ (IFN-γ) production, loss of Blimp-1 prevented GzmB expression in response to IL-2, suggesting two independent programs required for polyfunctionality of tumor-reactive CD4^+^ T cells. Our findings underscore the role of Treg cells, IL-2, and Blimp-1 in controlling the differentiation of cytotoxic CD4^+^ T cells and offer a pathway to enhancement of anti-tumor activity through their manipulation.

## Highlights

• Tumor-infiltrating CD4^+^ T cells exhibit T helper and cytotoxic features
• Treg cells limit IL-2 availability vital for the acquisition of cytotoxic features
• T-bet is required for IFN-γ, but not GzmB, expression in CD4^+^ TILs
• Blimp-1 is required for GzmB expression in response to IL-2 in CD4^+^ TILs

## Graphical Abstract

**Figure:**
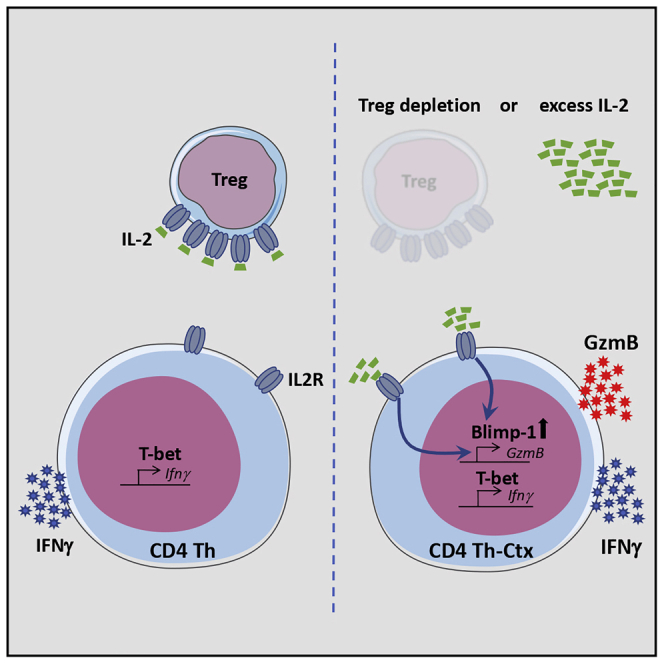

## Teaser

Śledzińska et al. show that upon depletion of regulatory T cells, a surplus of interleukin-2 in the tumor microenvironment supports acquisition of cytotoxic activity by T helper cells orchestrated by the transcription factor Blimp-1. These polyfunctional CD4^+^ T cells exhibit potent anti-tumor activity in an adoptive transfer setting with therapeutic implications.

## Introduction

Shortly after the definition of the classical T helper (Th) type 1 (Th1) and type 2 (Th2) lineages (Mosmann et al., 1986), it was reported that mycobacterial antigens could induce the development of cytotoxic CD4^+^ T cells (Mustafa and Godal, 1987, Ottenhoff et al., 1988). Such cytotoxic CD4^+^ T cells are found in both mice and humans in a wide range of pathological conditions (Juno et al., 2017), including murine cancer models where melanoma-reactive CD4^+^ T cells acquire cytotoxic activity and eliminate large transplantable and spontaneous mouse melanoma tumors (Quezada et al., 2010, Xie et al., 2010). Similarly, NY-ESO-1-specific CD4^+^ T cells isolated from melanoma patients are able to lyse melanoma cells expressing the cognate antigen. Moreover, the number of these cells in the blood increases after treatment with ipilimumab (αCTLA-4) (Kitano et al., 2013).

Several attempts have been made to define a set of surface markers that separate cytotoxic CD4^+^ T cells from other Th subsets, but there is no consensus as to whether such markers exist. Indeed, it is now widely accepted that CD4^+^ T cell lineages exhibit a degree of plasticity, with cells simultaneously expressing markers of more than one Th lineage and retaining the ability to switch phenotypes during their lifespan (DuPage and Bluestone, 2016). In keeping with this, granzyme B (GzmB)-secreting cytotoxic CD4^+^ T cells exhibit activation markers, cytokines, and transcription factors associated with different Th subsets (Takeuchi and Saito, 2017, Tian et al., 2016). Perforin (*PFR1*)- expressing human CD4^+^ T cells produce tumor necrosis factor α (TNF-α), interferon-γ (IFN-γ), and granzyme A (GzmA) (Appay et al., 2002).

The transcription factors involved in the differentiation of cytotoxic CD4^+^ T cells *in vivo* remain unclear. T-bet (*Tbx21*) and Eomes are potential candidates due to their well-established role in controlling Th1 responses and inducing *Gzmb* and *Prf1* expression in CD8^+^ T and natural killer (NK) cells (Evans and Jenner, 2013, Glimcher et al., 2004). T-bet also directly binds and activates *GZMB*, *PRF1*, and *NKG7* in CD4^+^ T cells *in vitro* (Kanhere et al., 2012). Studies in an adenovirus infection model showed that the cytotoxic program does not correlate with T-bet or Eomes expression and instead is in direct opposition to the Bcl6-driven follicular helper T (Tfh) cell differentiation program (Donnarumma et al., 2016). These virus-induced cytotoxic cells also exhibit higher expression of *Prdm1*, encoding the transcriptional repressor Blimp-1, previously shown to inhibit *Bcl6* and *Tcf7* expression in CD4^+^ T cells (Choi et al., 2015, Fu et al., 2017, Johnston et al., 2009, Wu et al., 2015).

The list of potential environmental factors regulating cytotoxic cell development ranges from T cell receptor (TCR) signal strength to members of the common gamma (cγ) chain cytokine family or IFN-α (Hua et al., 2013). *In vitro*, exogenous interleukin-2 (IL-2) increases the lytic potential of CD4^+^ T cells in response to low antigen dose (Brown et al., 2009), and IL-2 is a potent inducer of perforin and GzmB in CD8^+^ T cells (Janas et al., 2005). IL-2 opposes the differentiation of Tfh cells by decreasing Bcl6 expression (Ballesteros-Tato et al., 2012), hence playing a role in controlling the Bcl6/Blimp-1/Tcf1 balance (Fu et al., 2017).

Here, we examined the environmental signals and transcription factors regulating the development of cytotoxic CD4^+^ T cells within tumors and in the context of immunotherapy. In the adoptive cell therapy (ACT) setting, melanoma-specific TCR transgenic CD4^+^ T cells produced both IFN-γ and GzmB within tumors, suggesting that these cells have both helper and cytotoxic activities (Th-ctx). Transcriptional analysis of Th-ctx melanoma-reactive CD4^+^ T cells revealed high *Prdm1* and *Tbx21* expression and decreased expression of Tfh signature genes. IL-2 was central to the acquisition of the cytotoxic program in CD4^+^ T cells, functioning in a Blimp-1-dependent manner, and independent of the Th1 transcriptional program. Our findings provide insight into the mechanisms and context supporting the acquisition of cytotoxic function by CD4^+^ T cells, with implications for immunotherapies.

## Results

### CD4^+^ TCR Transgenic T Cells Acquire a Polyfunctional Th-Cytotoxic Phenotype upon Transfer into Tumor-Bearing Lymphopenic Mice

**Figure 1:**
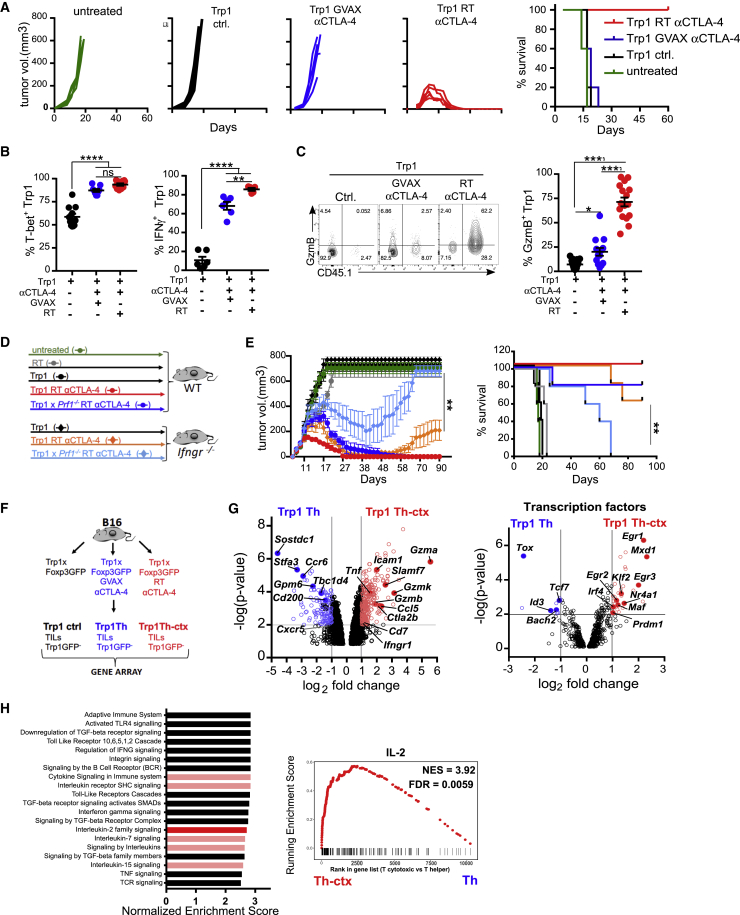
Tumor-Reactive CD4^+^ T Cells Acquire Cytotoxic Phenotype following Lymphopenia-Induced Expansion (A–C) B16-tumor-bearing mice were left untreated or treated with Trp1 cells (Trp1 ctrl), Trp1 + GVAX + αCTLA-4, or RT + Trp1 + αCTLA-4 as per Figure S1A. (A) Tumor growth and survival (N = 5/group). (B) T-bet and IFN-γ expression by Trp1 TILs (N = 10–11/group in 2 experiments) and (N = 5–6/group in two experiments), respectively. (C) Representative plot and quantification of GzmB expression by Trp1 TILs (N = 13–17/group in four experiments). (D and E) WT and *Ifngr1*^−/−^ mice bearing B16 tumors were left untreated or treated with Trp1 or Trp1xPrf-1^−/−^ cells alone or in combination with RT + αCTLA-4. (E) Tumor growth and survival (N = 5/group). (F–H) Foxp3^−^ Trp1 cells were sorted from B16 tumors from mice treated with GVAX + αCTLA-4 or RT + αCTLA-4 as per Figure S1D. (G) Total differential gene expression between Th Trp1 cells and Th-ctx Trp1 cells (p ≤ 0.01) and differentially expressed transcription factors between Trp1 Th-ctx cells and Trp1 Th cells (p ≤ 0.01). (H) Reactome pathway enrichment analysis of immune-system-related pathways and cytokine signaling pathways (red) (NES > 2, p < 0.05). Right panel: gene set enrichment analysis of IL-2-dependent genes (Castro et al., 2012; GEO: GSE39110). All quantification plots show mean ± SEM (one-way ANOVA).

Upon transfer into tumor-bearing lymphodepleted animals, melanoma-reactive tyrp-1-specific TCR transgenic CD4^+^ T cells (Trp1 cells) produce IFN-γ, TNF-α, and GzmB and acquire potent cytotoxic activity *in vitro* and *in vivo* (Quezada et al., 2010, Xie et al., 2010). To confirm whether this activity was specific to the Trp1 TCR or driven by therapeutic modality, we analyzed the activity of Trp1 cells in the context of host lymphodepletion combined with αCTLA-4 treatment or in response to a granulocyte-macrophage colony-stimulating factor (GM-CSF)-expressing tumor cell based vaccine (GVAX) combined with αCTLA-4, which also induces effective Trp1 cell activation and IFN-γ secretion *in vivo* (Simpson et al., 2013). B16 tumor-bearing mice were left untreated or treated at day 8 with total body irradiation (RT) + Trp1 + αCTLA-4, Trp1 + GVAX + αCTLA-4, or Trp1 cells in the absence of irradiation or vaccine as an additional control (referred to as control treatment [Trp1 ctrl.]) (Figure S1A). Transfer of Trp1 cells into irradiated hosts in combination with αCTLA-4 promoted rejection of large, established tumors in all treated mice, whereas Trp1 + GVAX + αCTLA-4 failed to drive complete responses (Figures 1A and S1B). To understand these different outcomes, we assessed the quantity and quality of Trp1 cell infiltrates following therapy. While both GVAX- and radiation-based therapies significantly enhanced Trp1 effector cell (CD4^+^Trp1^+^Foxp3^−^) proliferation within tumors, irradiation gave the largest, most significant increases in Trp1 effector numbers and ΔT effector (Teff)/Regulatory T (Treg) cell ratio compared to Trp1 monotherapy (Figure S1B). Both treatments (RT + Trp1 + αCTLA-4 and GVAX + Trp1 + αCTLA-4) induced high levels of T-bet and IFN-γ by tumor-infiltrating Trp1 cells (Figure 1B), suggesting acquisition of a Th1-like differentiation program. In contrast, only Trp1 CD4^+^ T cells primed in the lymphopenic environment (RT + Trp1 + αCTLA-4) increased GzmB expression, revealing a polyfunctional Th and cytotoxic phenotype (Figure 1C). TNF-α and IL-2 followed a similar pattern, with the highest levels observed in Trp1 expanded in lymphodepleted mice (Figure S1C; data not shown). GVAX-expanded Trp1 cells showed only a Th phenotype, with no significant increase in GzmB (from this point referred to as Trp1 Th). In keeping with the production of GzmB, Trp1 cells expanded in lymphopenic hosts specifically killed B16 tumor cells *in vitro* (Figure S1D). To determine the role of both helper and cytotoxic activities of Trp1 cells in tumor rejection, we transferred either Trp1 or perforin-1-deficient Trp1 cells (*Prf1*^−*/*−^Trp1) (Figure S1E) into wild-type (WT) or *Ifngr1*^−*/*−^ hosts combined with radiation and αCTLA-4 (Figure 1D). *Prf1*^−*/*−^Trp1 cells have reduced cytotoxicity (Kägi et al., 1994), while IFN-γR-deficient myeloid cells are less able to support Th-1 differentiation *in vivo* (Tau and Rothman, 1999). While WT recipients treated with *Prf1*^−*/*−^Trp1 cells grew larger tumors than mice treated with Trp1 cells (p < 0.01, between days 13 and 23), both treatments promoted rejection of established tumors. IFN-γR-deficient recipients treated with Trp1 + RT + αCTLA-4 showed partial tumor control followed by relapse. *Prf1*^−*/*−^Trp1 cells transferred to IFN-γR-deficient were unable to control tumor growth (Figures 1E and S1F), confirming that both Th1 and cytotoxic activities of Trp1 cells (Trp1 Th-ctx) are critical for maximal tumor control. We thus focused on investigating the molecular and environmental factors underpinning acquisition of cytotoxic activity by CD4^+^ T cells.

To gain insight into the molecular processes distinguishing Trp1 Th-ctx cells from Trp1 Th cells, we performed gene expression profiling on Trp1 Foxp3^−^ cells isolated from tumor and draining lymph nodes (dLNs) 8 days after treatment initiation (Figures 1F and S1G). Comparison of Trp1 Th-ctx cells to Trp1 Th cells isolated from tumors identified 382 differentially expressed genes (p < 0.01 and log2 fold change ≥2) (Table S1). *Gzmb*, *Gzma*, *Gzmk*, *Icam1*, and *Tnf* were found to be among the most increased genes in Trp1 Th-ctx compared to Trp1 Th cells, in keeping with our prior phenotypic analyses. We also observed higher expression of genes previously reported to be increased in cytotoxic CD4^+^ T cells recognizing viral antigens (Donnarumma et al., 2016), such as *Ccl5*, *Ctla2b*, and *Cd7*. There was, however, no significant difference in *Prf1* expression between the two conditions (Figure S1H).

Transcription factor genes increased in Trp1 Th-ctx cells (Table S2) included those belonging to the Kruppel-like factor family (*Klf2*, *Klf7*, and *Klf10*), of which Klf2 is known to promote T-bet and Blimp-1 expression (Lee et al., 2015), as well as transcription factors with established roles in shaping CD4^+^ T cell fate, including *Maf*, *Irf4*, *Prdm1*, and *Egr1* (Fu et al., 2017, Zhu and Paul, 2010) (Figure 1G). When analyzing the genes with significantly increased expression in GVAX-expanded Trp1 Th cells compared to Trp1 Th-ctx cells, we identified a set of genes previously reported to be associated with Tfh cells or natural Th21 cells, including *Sostdc1*, *Stfa3*, *Tox*, *Ccr6*, *Tcf7* (encoding TCF-1), *Gpm6b*, and *Cd200* (Marnik et al., 2017, Choi et al., 2015) (Figure 1G). While the genes highly expressed in Th-ctx cells do not specifically match a single defined CD4^+^ helper lineage (i.e., Th1, Th2, Th17, and Th21), we noted that many differentially expressed genes are regulated by Blimp-1, including *Socs1*, *Slamf1*, *Grap2*, *Maf*, *Ctla4*, and *Il10* (Bankoti et al., 2017). *Tbx21* and other master regulators were not differentially expressed between Th and Th-ctx cells; instead, *Tbx21* expression was increased in both conditions in comparison to control Trp1 cells, consistent with our flow cytometry analyses (Figure S1H).

Reactome pathway analysis revealed an increased expression of genes related to the apoptosis/survival pathway, Toll-like receptor activation, and cytokine signaling, including Cγ chain receptor signaling pathways, in Trp1 Th-ctx cells in comparison to Trp1 Th tumor infiltrating lymphocytes (TILs) (Figure 1H). Consistent with the increased expression of Cγ chain cytokine signaling genes, gene set enrichment analysis (GSEA) showed enrichment of IL-2 responsive genes (Castro et al., 2012) in Th-ctx conditions (Figure 1H). There were no significantly enriched pathways directly related to the immune system in Trp1 Th cells in comparison to Th-ctx cells (Figure S1I).

Taken together, these data suggest that both therapeutic modalities (GVAX + αCTLA-4 and RT + αCTLA-4) promote differentiation of Trp1 T cells into a core polyfunctional Th cell phenotype with marked Th1-like characteristics. However, while GVAX + αCTLA-4 favored a Th follicular-like signature in tumor-infiltrating Trp1 cells, RT + αCTLA-4 supported the acquisition of additional transcriptional programs associated with cytotoxicity (Th-ctx).

### Endogenous IL-2 Drives GzmB Expression in Both Murine and Human CD4^+^ T Cells *In Vitro*

**Figure 2:**
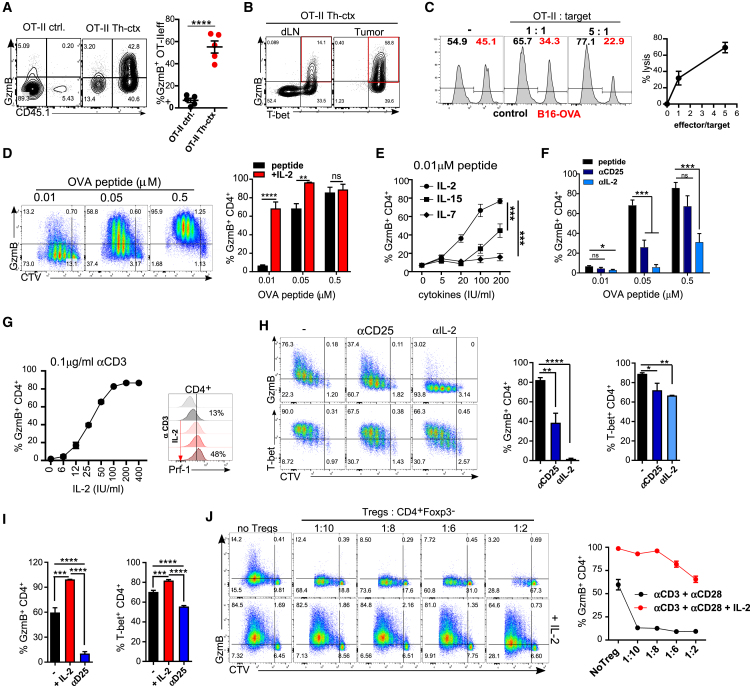
IL-2 Drives GzmB Expression in Both Murine and Human CD4^+^ T Cells *In Vitro* (A and B) OT-II cells were transferred to mice bearing B16-OVA tumors (as per Figure S2A) alone or in combination with RT + αCTLA-4. (A) Representative plots and quantification of GzmB-expressing OT-II TILs (N = 5/group). (B) Representative plots showing expression of GzmB and T-bet by OT-II cells in the Th-ctx condition. (C) OT-II cytotoxicity assay. Representative plots and quantification of specific lysis are shown. (D–F) Cell trace violet (CTV)-labeled OT-II cells were stimulated with indicated concentrations of OT-II peptide to asses GzmB expression within proliferating cells in the following conditions: (D) addition of IL-2 (two independent experiments), (E) addition of indicated cytokines, and (F) addition of 5 μg/ml of indicated antibodies. (G and H) CTV-labeled murine polyclonal CD4^+^ T cells were stimulated with (G) αCD3 and IL-2 to assess expression of GzmB in proliferating CD4^+^ T cells. A representative plot of perforin expression is also shown. (H) αCD3 and αCD28 and indicated antibodies. Representative plots and quantification of GzmB and T-bet-expressing cells (data are representative of two independent experiments). (I and J) CTV-labeled human polyclonal naive CD4^+^ T cells stimulated with αCD3 and αCD28. (I) After 24 h, either IL-2 or αCD25 antibody was added. Quantification of GzmB- and T-bet-expressing CD4^+^ T cells (cumulative data of two independent experiments). (J) Indicated ratios of autologous Treg cells added with or without IL-2. Representative plots and quantification of GzmB -expressing CD4^+^ T cells are shown. All quantification plots show mean ± SEM (one-way ANOVA) (B, two-way ANOVA).

To determine whether the acquisition of the polyfunctional Th1 and cytotoxic phenotype was specific to the Trp1 system, we repeated these experiments in mice bearing B16 tumors expressing ovalbumin (B16-OVA) and treated with OVA-reactive OT-II TCR Tg CD4^+^ T cells (Figure S2A). OT-II cells transferred into irradiated B16-OVA-bearing mice expressed GzmB and promoted rejection of established tumors consistent with the Trp1 model (Figures 2A and S2A). GzmB^+^ OT-II cells also co-expressed T-bet (Figure 2B) and were able to directly kill B16-OVA tumor in a GzmB-dependent manner (Figures 2C and S2B). These data suggest that acquisition of Th-ctx cell phenotype is not unique to the Trp1 TCR.

Increased expression of genes associated with Cγ chain cytokine signaling and the response to IL-2 in Trp1 Th-ctx is consistent with the cytokine milieu induced by lymphodepletion (Williams et al., 2007) and could offer mechanistic insights into the acquisition of cytotoxic activity by tumor-reactive CD4^+^ T cells *in vivo*. We therefore evaluated the potential contribution of Cγ receptor cytokines to the gain of GzmB in tumor-reactive CD4^+^ T cells. Briefly, Trp1 and OT-II TCR transgenic T cells were stimulated 3 days *in vitro* with different concentrations of cognate antigen, in the presence or absence of IL-2, IL-7, or IL-15. Both Trp1 and OT-II T cells increased GzmB production with increasing antigen dose, while exogenous IL-2 augmented GzmB at lower antigen concentrations (Figures 2D and S2C). IL-2 was the most potent inducer of GzmB at low antigen concentration, followed by IL-15 and, at a much lower level, IL-7 (Figure 2E). Endogenous IL-2 was critical for GzmB production *in vitro*, as both IL-2 neutralization and CD25 receptor blockade reduced GzmB expression by transgenic cells in response to antigen (Figures 2F and S2C).

The high levels of T-bet in Trp1 and OT-II Th-ctx cells led us to evaluate its possible association with IL-2 and GzmB expression. While IL-2 deprivation reduced T-bet expression by *in*-*vitro*-activated OT-II cells (Figure S2D), the impact on T-bet was less marked than the impact on GzmB, suggesting that these two pathways may not be directly linked. Further validation was obtained using polyclonal mouse CD4^+^ T cells stimulated with αCD3 + IL-2. Increasing amounts of IL-2 significantly augmented both GzmB and perforin expression in CD4^+^ T cells (Figure 2G). Furthermore, endogenous IL-2 was critical for the increase of GzmB expression in polyclonal CD4^+^ T cells stimulated with αCD3 and αCD28, as IL-2 neutralization diminished GzmB expression with minimal impact on T-bet (Figure 2H). Consistent with the mouse data, stimulation of naive human CD4^+^ T cells with αCD3 and αCD28 resulted in GzmB expression in 60% of the cells. This increased to 95% upon addition of exogenous IL-2. In contrast, blockade of IL-2R signaling with αCD25 (basiliximab) significantly reduced GzmB to untreated control levels (Figure 2I).

IL-2 deprivation is utilized by Treg cells to suppress T-cell-mediated immunity, primarily impacting proliferation and survival (Sakaguchi et al., 2008). To determine whether Treg cells also suppress acquisition of cytotoxic potential by CD4^+^ T cells, we activated purified human naive CD4^+^ T cells and co-cultured them with different ratios of autologous Treg cells. Low numbers of Treg cells (1:10 Treg/Teff cells) significantly suppressed GzmB expression, whereas T-bet was only partially affected (Figure 2J; data not shown). A higher ratio of Treg:Teff cells was needed in order to effectively suppress CD4^+^ Teff cell proliferation *in vitro* (Figures 2J and S2E). Exogenous IL-2 was able to revert both effects, increasing GzmB production and proliferation even at the highest Treg/Teff cell ratios. These data suggest that endogenous IL-2 drives GzmB expression on CD4^+^ Teff cells while Treg cells negatively control this process, potentially through IL-2 competition.

### Endogenous IL-2 Contributes to an Increase in GzmB Expression by Adoptively Transferred Tumor-Reactive T Cells *In Vivo*

**Figure 3:**
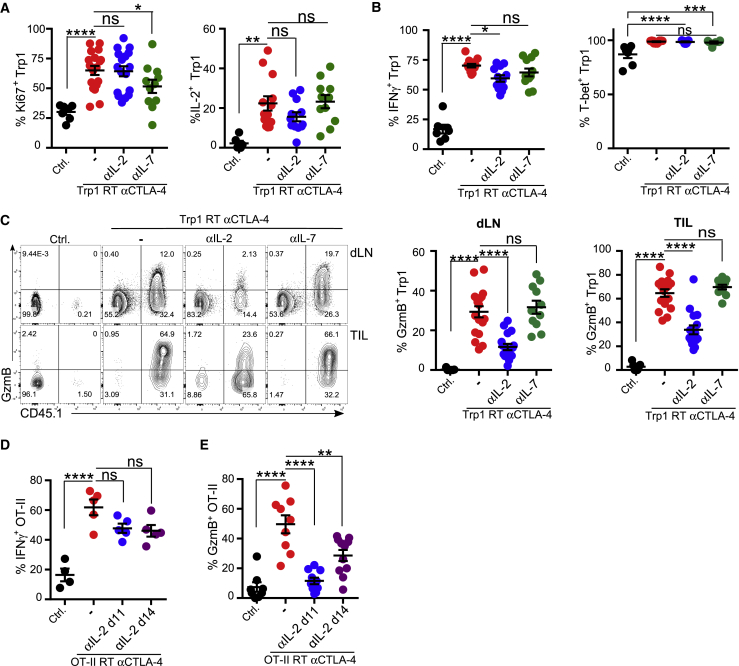
CD4 TILs in the Th-ctx Condition Reduce GzmB Expression but Retain the Th1 Phenotype upon IL-2 Neutralization (A–C) B16-tumor-bearing mice were left untreated (Ctrl) or received RT + Trp1 + aCTLA-4 (Th-ctx) with or without αIL-2 or αIL-7 (see Figure S3A). Quantification of (A) Ki-67- (N = 7–18/group) and IL-2-expressing Trp1 cells (N = 7–13/group), (B) T-bet- and IFN-γ- expressing Trp1 cells (N = 7–13/group in two independent experiments), and (C) GzmB expression in Trp1 (CD45.1^+^) and endogenous CD4^+^ T cells in LN and tumors (N = 7-13/group in three experiments). (D and E) B16-OVA-tumor-bearing mice were treated as shown in Figure S3D. Tumors and dLNs were isolated at day 18 post-tumor inoculation for analysis. (D) Quantification of IFN-γ- (N = 5/group) and (E) GzmB-expressing cells within OT-II cells (N = 8–11/group in two experiments). All quantification plots show mean ± SEM (one-way ANOVA).

We sought to determine whether IL-2 controls GzmB expression in adoptively transferred tumor-reactive CD4^+^ T cells *in vivo*. Briefly, B16-bearing mice received Trp1 cells alone (ctrl) or with irradiation and αCTLA-4 (Trp1 + RT + αCTLA-4) in the presence or absence of an αIL-2 neutralizing antibody. An additional group of mice received an αIL-7 neutralizing antibody to rule out its potential role in GzmB regulation *in vivo*, as this is relevant for CD8^+^ T cells (Li et al., 2011). IL-2 or IL-7 neutralization was started 3 days after adoptive transfer to allow the initial expansion of transferred T cells (Figure S3A). IL-2 neutralization did not decrease the frequency of Ki67-expressing cells within the Trp1 cells in contrast to αIL-7 treatment, which significantly reduced CD4^+^ Trp1 cell proliferation (Figure 3A). Tumor-infiltrating Trp1 Th-ctx cells expressed high levels of IL-2, with neither αIL-2 nor αIL-7 treatment impacting its expression (Figure 3A). As expected, IL-2 neutralization resulted in decreased expression of the high-affinity IL-2 receptor CD25 on activated CD4^+^ T cells (Figure S3B). When assessing effector function, we observed a small decrease in IFN-γ expression upon IL-2 neutralization, although T-bet expression was not significantly reduced (Figure 3B). Among the cytokines we analyzed in these settings, GM-CSF protein expression was partially decreased by IL-2 neutralization in the tumor, but not in dLNs (Figure S3C; data not shown). The frequency of Trp1^+^GzmB-expressing cells was, however, significantly reduced in αIL-2-treated mice, both in the dLN and in the tumor (Figure 3C). αIL-7 did not impact GzmB expression by Trp1 cells, recapitulating the *in vitro* data (Figure 3C). Similar results were obtained in the B16-OVA tumor and OT-II model (Figure S3D). In these experiments, αIL-2 treatment started at two different time points post-OT-II T cell transfer based on previous experiments defining the temporal profile of GzmB expression (data not shown). IL-2 neutralization at both time points caused a moderate decrease in IFN-γ expression by OT-II cells without affecting T-bet expression (Figure 3D; data not shown). The frequency of OT-II cells with lytic potential was significantly decreased when the αIL-2 treatment started 3 or 5 days post-transfer (Figure 3E; data not shown). These findings support a critical role for IL-2 in the acquisition and maintenance of a Th-ctx phenotype in the adoptive transfer setting.

### Increased IL-2 Availability after Treg Cell Depletion Contributes to Shaping Th Cell Phenotype within Tumors

**Figure 4:**
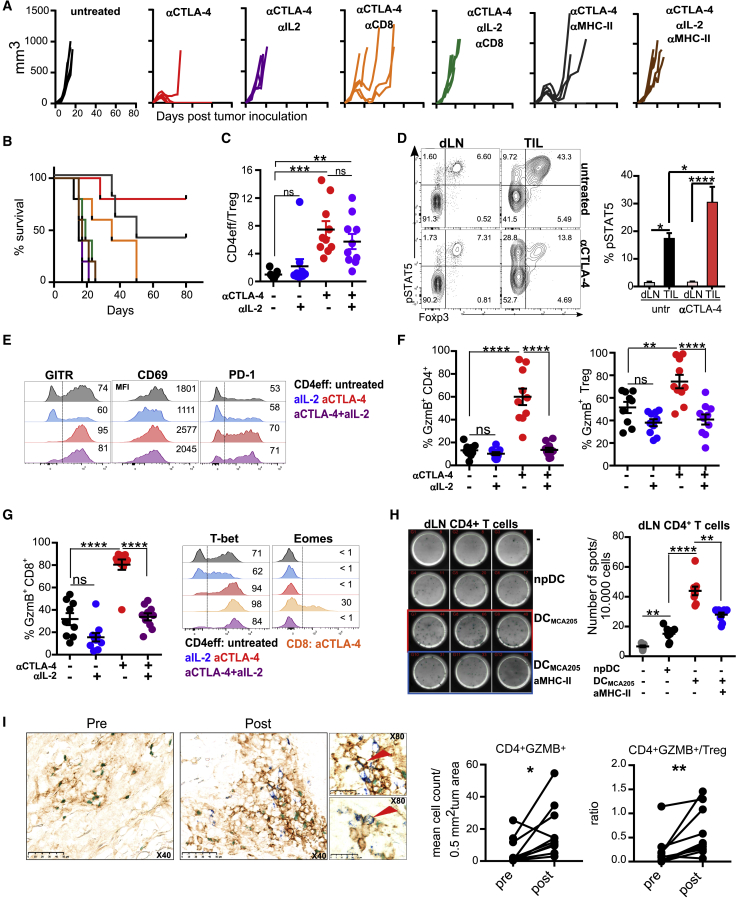
Increased IL-2 Availability after Treg Cell Depletion Contributes to Shaping the Th Cell Phenotype within Tumors (A and B) MCA205-tumor-bearing mice were treated with αCTLA-4 on days 6, 9, and 12 alone or with αIL-2, αCD8, and αMHC class II on days 6, 9, 12, and 15 after tumor implantation. (A) Individual tumor growth curves and (B) cumulative survival are shown. (C–G) MCA205-tumor-bearing mice were treated with αCTLA-4, αIL-2, or combination as in (A). TILs and dLNs were isolated 13 days post-tumor inoculation. (C) CD4eff/Treg cells in tumors (N = 10/group in two independent experiments). (D) TILs and dLNs from MCA205-bearing mice were re-stimulated with IL-2. Representative plots of pSTAT5 expression in CD4^+^ T cells and quantification of pSTAT5-expressing CD4eff T cells are shown (N = 5/group in two independent experiments). (E) Expression of GITR, CD69, and PD-1 by CD4eff TILs. Representative plots are shown with mean percentage of expression or mean fluorescence intensity (N = 5–10/group; two independent experiments). (F) Quantification of GzmB-expressing cells within CD4eff TILs and Treg TILs (N = 10/group in two independent experiments. (G) Quantification of GzmB-expressing cells within CD8 TILs and expression of Eomes and T-bet by CD4eff and CD8 TILs. Representative plots are shown with mean percentage of expression or mean fluorescent intensity (N = 10/group; two independent experiments). (H) dLN-infiltrating CD4^+^ T cells were cultured unstimulated or stimulated with MCA205-pulsed dendritic cells (DCs) or empty (np) DCs on αGzmB-coated ELISPOT plate for 24 h. Numbers represent GzmB spots per 10,000 responding CD4^+^ T cells. Graphical representation and quantification are shown. (I) Immunohistochemical analysis of GzmB expression by CD4^+^ T cells in human melanoma. Representative plots pre- and post-therapy are shown, with CD4 staining in brown, FOXP3 in green, and GZMB in blue. Quantification of CD4^+^GZMB^+^ cells within tumor pre- and post-treatment and ratio of CD4eff to CD4^+^FOXP3^+^ cells are shown (n = 10 patients, Wilcoxon matched-pairs signed rank test, one tailed). All other quantification plots show mean ± SEM (one-way ANOVA).

To determine whether our findings were relevant in the context of non-TCR transgenic T cells, we evaluated the role of IL-2 in the acquisition of cytotoxic activity by CD4^+^ T cells in MCA205 sarcoma, which is known to respond to αCTLA-4 monotherapy. To test whether IL-2 was necessary for the anti-tumor activity driven by αCTLA-4, we inoculated WT mice with MCA205 and treated with αCTLA-4 in the presence or absence of a neutralizing αIL-2. In keeping with previous studies (Hannani et al., 2015), neutralization of IL-2 abolished the anti-tumor activity of αCTLA-4. Furthermore, both CD4^+^ and CD8^+^ T cells were required for αCTLA-4-mediated MCA205 tumor control (Figures 4A and 4B). To identify the role of IL-2 during αCTLA-4-mediated CD4^+^ T cell activation and differentiation, MCA205-bearing mice were treated as above, and tumor size was measured to ascertain the impact of the different treatments prior to assessment of CD4^+^ T cell differentiation within tumors (Figure S4A). As IL-2 plays an important role in Treg cell homeostasis and function (Ye et al., 2018), we compared the number of Treg cells within the CD4^+^ TIL compartment across all conditions. αIL-2 treatment alone significantly reduced number of Treg cells in tumors, which was further decreased by αCTLA-4, consistent with its Treg-cell-depleting activity (Figure S4B). IL-2 neutralization slightly reduced the number of CD4eff TILs, whereas αCTLA-4 treatment increased their numbers, giving a significantly increased Teff/Treg ratio only in αCTLA-4- and αCTLA-4- + αIL-2-treated tumors (Figure 4C).

We assessed the expression and activation of IL-2 signaling pathway components in CD4^+^ TILs and dLN cells in the MCA205 tumor model. The frequency of CD25- and CD122-expressing CD4^+^ effector T cells was increased in αCTLA-4 treated tumors. IL-2 neutralization reduced the proportion of CD25^+^ CD4^+^ T cells, but not the frequency of CD122-expressing CD4^+^ T cells (Figure S4C). To determine if the increased percentage of CD25^+^CD4^+^eff TILs translated into an increased frequency of CD4eff cells with elevated IL-2 signaling, MCA205 TILs and lymph node (LN) cells were re-stimulated with IL-2, and phosphorylation of STAT5 was measured. An increase in STAT5 phosphorylation confirmed activation of the IL-2 pathway in CD4^+^ effector TILs in both untreated and αCTLA-4-treated tumors in comparison to CD4^+^ T cells in the LN (Figure 4D). Further analysis of T cell activation markers revealed increased expression of CD69, CD44, GITR (Glucocorticoid-Induced TNFR-Related Protein) and CD38 upon αCTLA-4 treatment, of which only GITR (McNamara et al., 2014) was decreased by IL-2 deprivation relative to controls. PD-1 expression was consistently increased and the negative co-stimulatory molecule CD101 decreased (Schey et al., 2016) in all αCTLA-4 treated groups regardless of IL-2 presence (Figures 4E and S4D). The fraction of GzmB-expressing CD4^+^ T cells was increased in the tumor following αCTLA-4 treatment in accordance with the TCR transgenic data. When αCTLA-4 was combined with αIL-2, however, the GzmB levels in TILs decreased to the level of untreated mice (Figure 4F). In contrast, NK cells showed no significant change, and GzmB expression by T cells in the dLN was negligible (Figure S4E). As in the ACT models, activated CD4^+^ T cells acquired a Th1-like phenotype, characterized by increased expression of T-bet and, in contrast to activated CD8^+^ T cells, no Eomes (Figures 4G and S4F). Importantly, we found that Th-ctx CD4^+^ T cells isolated from αCTLA-4-treated MCA205 tumors were able to release GzmB when co-cultured with MCA205-loaded dendritic cells in a major histocompatibility complex class II (MHC class II)-dependent manner (Figures 4H and S4I**).** While IL-15 was able to increase GzmB expression by activated CD4^+^ T cells *in vitro*, IL-15 neutralization *in vivo* in MCA205-bearing αCTLA-4 treated mice had no impact on GzmB (Figure S4H). Thus IL-2 is critical for GzmB expression by endogenous tumor-infiltrating CD4^+^ T cells with little impact on other features of CD4^+^ T cell activation and differentiation in response to αCTLA-4.

We next sought to determine whether a similar relationship between Treg cell numbers and increased GzmB expression in CD4^+^ T cells could be found in tumors from patients treated with αCTLA-4. We used triple-color immunohistochemistry to evaluate Foxp3 and GzmB expression in CD4^+^ TILs in patients with advanced melanoma prior to therapy and 3 weeks post-treatment with ipilimumab and melphalan. This model demonstrated clinical efficacy with a 10-fold increase in CD4^+^ T cells on average in the tumor after treatment (Ariyan et al., 2018). We observed a significant increase in the number of GzmB^+^CD4^+^Foxp3^−^ T cells post-therapy. Furthermore, a significant increase in the ratio of GzmB^+^CD4^+^Foxp3^−^ to CD4^+^ Foxp3^+^ cells was also observed post-treatment (Figure 4I), suggesting an inverse relationship between Treg cell frequency and GzmB expression in tumor-infiltrating CD4^+^ T cells. Together, the mouse and human data support a model in which Treg cells control acquisition of cytotoxic activity by tumor-infiltrating CD4^+^ T cells.

### Treg Cell Depletion in the Absence of CTLA-4 Blockade Drives GzmB Expression by CD4^+^ T Cells

**Figure 5:**
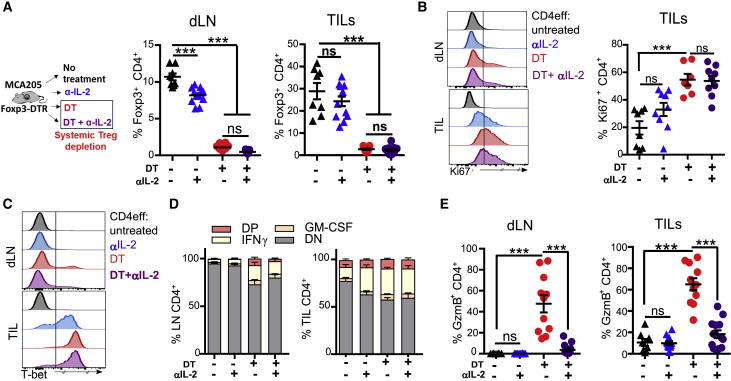
Treg Cell Depletion in the Absence of CTLA-4 Blockade Drives GzmB Expression by CD4^+^ T Cells (A–E) MCA205-bearing Foxp3^DTR^ mice were treated with DT alone or in combination with αIL-2 (schema in Figure S5F) from day 6 post-tumor inoculation. (A) Schema and quantification of Treg cells within CD4 T cells in dLNs and TILs (N = 10/ group in two experiments). Expression of (B) Ki67 (C) T-bet by CD4 TILs (representative plots and quantification). (D) Quantification of CD4^+^ IFN-γ- and GM-CSF-expressing cells (N = 10/group in two experiments). (E) Quantification of CD4^+^ GzmB-expressing cells within dLNs and TILs (N = 10/group in two experiments). All quantification plots show mean ± SEM (one-way ANOVA).

To further explore the hypothesis that increased IL-2 availability is a crucial factor in the differentiation of CD4^+^ Th-ctx cells after αCTLA-4-mediated Treg cell depletion, we used Foxp3^DTR^ mice to allow specific depletion of Treg cells without CTLA-4 blockade. We challenged Foxp3^DTR^ mice with MCA205 tumors and treated them with diptheria toxin (DT) with or without αIL-2 (Figure S5A). In contrast to αCTLA-4-mediated Treg depletion, which is tumor specific (Simpson et al., 2013), DT depletes Treg cells systemically (Kim et al., 2007) (Figure 5A), promoting general activation of CD4^+^ and CD8^+^ T cells in both dLNs and tumors. Combining DT-mediated Treg cell depletion with αIL-2 had little impact on Ki67 (Figures 5B and S5B), T-bet, IFN-γ, and GM-CSF expression in CD4^+^ T cells in either LN or tumor (Figures 5C, 5D, and S5C). Treg cell depletion promoted GzmB upregulation in TILs and draining LN T cells, which was abrogated (in CD4^+^ and CD8^+^ T cells) by IL-2 neutralization (Figures 5E and S5D). These data suggest that increased IL-2 as a consequence of a lower Treg cell to CD4^+^eff cell ratio is a key factor contributing to acquisition of a cytotoxic phenotype by CD4^+^ T cells *in vivo*.

### *In Vivo* Acquisition of a Cytotoxic Phenotype by CD4^+^ TILs and Tumor Rejection Are Independent of T-bet Expression

**Figure 6:**
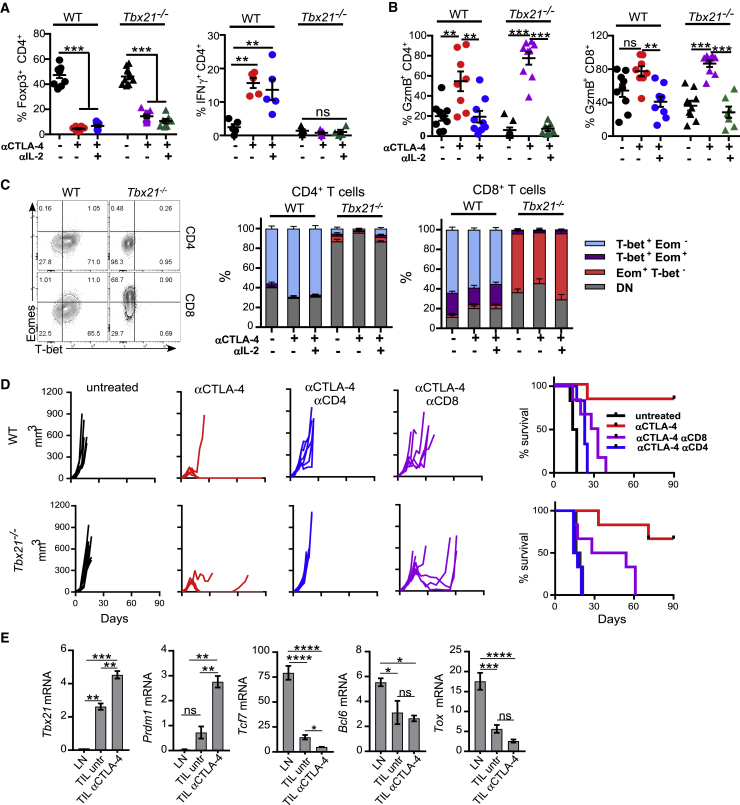
T-bet Is Not Required for CTLA-4-Mediated Rejection of MCA205 Sarcoma (A–C) WT and *Tbx21*^−/−^ MCA205-tumor-bearing mice were treated with αCTLA-4, αIL-2, or their combination. TILs and dLNs were isolated 13 days post-tumor inoculation for analysis of (A) Treg cells (N = 7–9/group in two independent experiments) and IFN-γ (N = 4–5/group) within CD4^+^ TILs and (B) GzmB-expressing cells within CD4eff and CD8^+^ TILs (N = 7–9/group in two independent experiments). (C) T-bet and Eomes expression by GzmB^+^ CD4eff and CD8^+^ TILs (N = 7–9/group in two experiments). (D) Tumor growth and survival in WT and *Tbx21*^−/−^ mice bearing MCA205 tumors and treated with αCTLA-4 alone or combined with depleting αCD8 or αCD4 antibodies on days 1, 3, 8, and 17 post-tumor implantation. (E) qRT-PCR for transcription factors in purified MCA205 CD4^+^Fopx3^−^ TILs and LNs at day 12 post-tumor inoculation (untreated versus αCTLA-4 treated mice). Results shown are expression relative to *Hprt1* expression using the 2^−ΔΔC(t)^ method (N = 6/condition). All quantification plots show mean ± SEM (one-way ANOVA).

In all analyzed conditions, *in vitro* and *in vivo*, CD4^+^GzmB^+^ T cells co-expressed the Th1 lineage-defining transcription factor T-bet. To investigate if T-bet had a dual role, controlling both Th1 and cytotoxic features of CD4^+^ T cells, we inoculated *Tbx21*^−*/*−^ and WT mice with MCA205 tumors followed by treatment with αCTLA-4 alone or in combination with neutralizing αIL-2. As expected, αCTLA-4-mediated Treg cell depletion in *Tbx21*^−*/*−^ mice failed to induce IFN-γ expression (Figure 6A; data not shown). However, WT and T-bet-deficient CD4^+^ and CD8^+^ TILs exhibited equal levels of GzmB in αCTLA-4 treated tumors (Figure 6B), suggesting that T-bet is not required for IL-2-mediated GzmB expression in CD4^+^ TILs.

The transcription factor Eomes can compensate for T-bet deficiency in CD4^+^ T cells (Yang et al., 2008). To exclude this, we compared Eomes expression in WT and *Tbx21*^−*/*−^ GzmB^+^ TILs. In contrast to T-bet-deficient GzmB^+^ CD8^+^ T cells, which express high amount of Eomes, *Tbx21*^−*/*−^ GzmB^+^ CD4^+^ T cells remained Eomes negative (Figure 6C), thus ruling out its potential involvement. To determine whether IFN-γ^−^ GzmB^+^ CD4^+^ T cells contribute to tumor control, WT and *Tbx21*^−*/*−^ mice were inoculated with MCA205 tumors and treated with αCTLA-4 (Figure 6D). Despite impaired IFN-γ production by T cells, *Tbx21*^−*/*−^ mice treated with αCTLA-4 were able to reject tumors at a similar rate to WT animals. Depletion of either CD8^+^ or CD4^+^ T cells underscored the relative contribution of each compartments. Critically, *Tbx21*^−*/*−^ mice depleted of CD8^+^ T cell were still able to promote tumor regression in 50% of the cases, whereas CD4 depletion resulted in complete loss of tumor control (Figure 6D). T-bet-deficient CD4^+^ T cells were able to control tumor growth even more effectively than WT CD4^+^ T cells in CD8 T-cell-depleted mice, supporting the relevance of CD4^+^GzmB^+^ T cells in tumor control induced by αCTLA-4.

Last, we analyzed the expression of other transcription factors potentially involved with CD4 cytotoxic activity and previously identified on the Trp1 gene array. Consistent with the Trp1 data, polyclonal Th-ctx cells infiltrating αCTLA-4-treated MCA205 tumors showed increased *Prdm1* and lower expression of *Tcf7* and *Tox* in CD4^+^ T cells relative to CD4^+^ TILs from untreated animals (Figure 6E). *Bcl6* expression was lower in TILs relative to LN CD4^+^ T cells regardless of the treatment. Together, these data suggest the cytotoxic activity of CD4^+^ TILs is independent of classical T-bet-dependent Th1 lineage programing.

### IL-2 Induces Cytotoxic CD4^+^ T Cell Differentiation via Both Blimp-1-Dependent and Blimp-1-Independent Mechanisms

**Figure 7:**
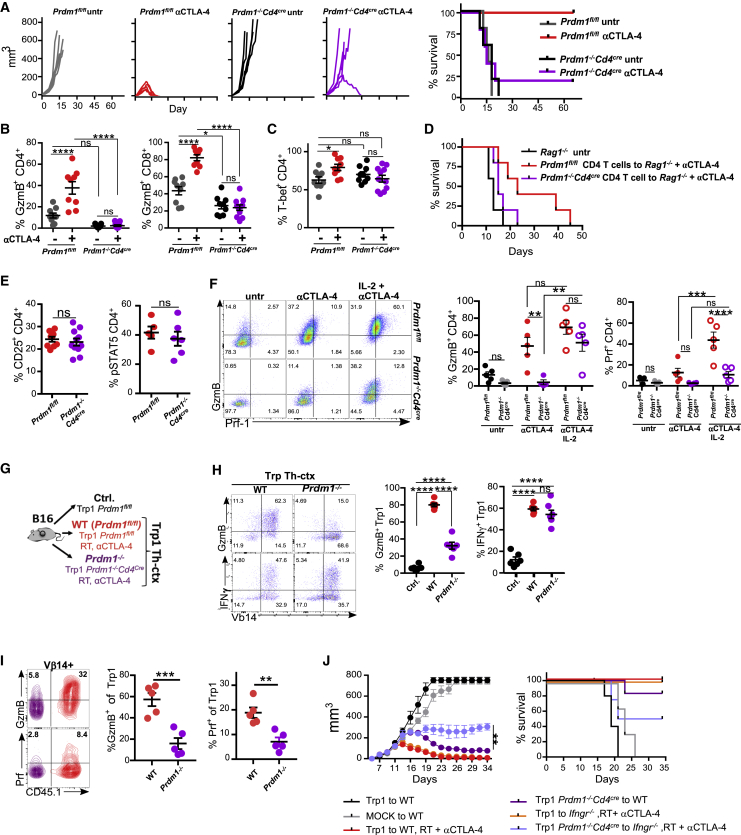
IL-2 Controls Cytotoxic CD4^+^ T Cell Differentiation in a Blimp-1-Dependent Manner (A) *Prdm1*^fl/fl^ and *Prdm1*^−/−^*Cd4*^*cre*^ mice bearing MCA205 tumors were treated or not with αCTLA-4 and monitored for tumor growth and survival. (B and C) TILs and dLNs were isolated at day 12 post-tumor inoculation for quantification of (B) GzmB-expressing cells within CD4eff and CD8 TILs and (C) T-bet-expressing cells within CD4eff and CD8 TILs (N = 9–11/group in two experiments). (D) Purified CD4^+^ T cells from dLNs and tumors from MCA205-bearing *Prdm1*^fl/fl^ and *Prdm1*^−/−^*Cd4*^*cre*^ mice were transferred to MCA205-bearing *Rag1*^−/−^ mice at day 3 post-inoculation followed by αCTLA-4. Overall survival is shown (N = 5/group). (E) Quantification of CD25- and IL-2-stimulated pSTAT5-expressing CD4eff TILs (N = 5–11 group in two experiments). (F) *Prdm1*^fl/fl^ and *Prdm1*^−/−^*Cd4*^*cre*^ MCA205 tumor-bearing mice were treated with αCTLA-4 alone or in combination with high-dose intratumoral IL-2. TILs and dLNs were isolated at day 12 post-tumor inoculation for quantification of GzmB- and Prf-1-expressing cells within CD4^+^ TILs (N = 5/group). (G) Purified CD4^+^ T cells from WT (*Prdm1*^fl/fl^) and *Prdm1*^−/−^*Cd4*^*cre*^ mice were transduced with Trp1 TCR-expressing vector and transferred to B16-bearing mice alone or in combination with αCTLA-4 + RT. (H) Representative plots and quantification of GzmB- and IFN-γ-expressing cells within Trp1 effector TILs (N = 6/group in two independent experiments). (I) Transduced WT and Blimp-1-deficient Trp1 cells co-transferred 1:1 to the same host. Representative plots and quantification of GzmB-and Prf-1-expressing cells within Trp1 TILs are shown (N = 5/group). (J) Transduced cells as in (H) were transferred at day 8 to B16-bearing WT or *Ifngr*^−/−^ mice alone or in combination with αCTLA-4 + RT. Cumulative tumor growth and survival are shown (N = 5/group). All quantification plots show mean ± SEM (one-way ANOVA) (J, two-way ANOVA).

Considering T-bet does not appear regulate GzmB expression and the high levels of *Prdm1* mRNA observed in CD4^+^ Th-ctx cells post-αCTLA-4 treatment, we explored whether Blimp-1 contributed to the acquisition of a cytotoxic phenotype by CD4eff T cells and to the overall anti-tumor activity of αCTLA-4. *Prdm1*^−*/*−^*Cd4*^*cre*^ and control *Prdm1*^fl/fl^ mice were inoculated with MCA205 cells and treated or not with αCTLA-4. *Prdm1*^−*/*−^*Cd4*^*cre*^ mice failed to control tumor growth after αCTLA-4 (Figure 7A), suggesting a critical role for this transcription factor during anti-tumor immunity *in vivo*. Characterization of TILs showed impaired expression of GzmB by CD4^+^eff and Treg cells in Blimp-1-deficient mice treated with αCTLA-4, whereas GzmB expression in CD8^+^ T cells was only partially reduced (Figures 7B and S6A). T-bet expression was not altered by Blimp-1 deletion in activated CD4^+^ T cells (Figure 7C), suggesting the Th1 program does not require Blimp-1, while the cytotoxic program (in both CD4^+^ and CD8^+^ T cells) depends on Blimp-1, which is critical for tumor control. To evaluate the role of Blimp-1 on CD4-driven tumor control, we isolated CD4^+^ TILs from MCA205 tumors and dLNs from αCTLA-4-treated *Prdm1*^fl/fl^ or *Prdm1*^−*/*−^*Cd4*^*cre*^ mice and transferred them to MCA205-bearing *Rag1*^−*/*−^ mice. Blimp-1-deficient CD4^+^ T cells showed reduced tumor control compared to WT CD4^+^ T cells (Figures 7D and S6B), supporting the relevance of Blimp-1 in CD4-mediated tumor control.

WT and Blimp-1-deficient CD4^+^ TILs in αCTLA-4-treated tumors exhibited equal levels of CD25 expression and STAT5 phosphorylation (Figure 7E), suggesting that failure to increase GzmB production was not due to deficient IL-2 signaling. To investigate the impact of IL-2 on GzmB expression by MCA205 TILs, *Prdm1*^−*/*−^*Cd4*^*cre*^ and *Prdm1*^*fl/fl*^ mice were inoculated with MCA205 cells and left untreated or treated with αCTLA-4 alone or combined with high-dose intratumoral IL-2. IL-2 increased the fraction of GzmB^+^ Blimp-1-deficent CD4^+^ and CD8^+^ TILs, but with a lower mean fluorescence intensity (MFI) than WT, suggesting lower expression per cell. Perforin expression by CD4^+^ and CD8^+^ TILs was not restored to WT levels on CD4^+^ TILs (Figures 7F and S6C). These data support an additional, Blimp-1-independent mechanism for regulation of GzmB expression by IL-2.

We next investigated if Blimp-1 deficiency affected the generation and function of Trp1 Th-ctx cells described at the beginning of this study. Purified WT (*Prdm1*^fl/fl^) and Blimp-1-deficient (*Prdm1*^−*/*−^) CD4^+^ T cells were transduced to express the Trp1 α and β TCR chains, with a mean transduction efficiency of 50% (data not shown). Trp1 WT and Blimp-1-deficient cells were transferred into B16-bearing mice with or without irradiation and αCTLA-4. As a control, mock-transduced WT CD4^+^ T cells were transferred into irradiated WT mice (Figures 7G and S6E). GzmB expression was reduced in *Prdm1*^−*/*−^Trp1 cells in comparison to WT Trp1 cells, but the proportion of Trp1 cells expressing TNF-α, T-bet, and IFN-γ was not different between Blimp-1-deficient and WT Trp1 cells (Figures 7H and S6F). Consistent with previous reports, Blimp-1-deficient Trp1 cells produced more IL-2 upon re-stimulation (Martins et al., 2008) (Figure S6F). *Prdm1*^−*/*−^Trp1 cells contained fewer cells positive for CD69, Lag3, and OX-40 than WT Trp1 cells (Figure S6G). These data suggest that IL-2-mediated GzmB upregulation by CD4^+^ T cells is, at least in part, regulated by Blimp-1 in the context of ACT. To control for the differences in the tumor microenvironment, we also co-transferred WT and *Prdm1*^−*/*−^Trp1 cells (ratio 1:1) to the same irradiated recipient. *Prdm1*^−*/*−^Trp1 cells exhibited lower levels of GzmB and Prf-1 than WT Trp1 cells, suggesting that the differences are intrinsic to Blimp-1-deficient cells (Figure 7I).

To assess the anti-tumor activity of *Prdm1*^−*/*−^Trp1 Th-ctx cells, we transferred WT and *Prdm1*^−*/*−^Trp1 cells into irradiated WT or *Ifngr1*^−*/*−^ B16-bearing recipients. We used *Ifngr1*^−*/*−^ recipients to evaluate the role of Blimp-1 in a model with impaired helper activity of Trp1 Th-ctx cells, as these mice cannot respond to IFN-γ produced by Trp1 Th-ctx cells. Mice treated with *Prdm1*^−*/*−^Trp1 cells, which produce less GzmB (Figure 7G), developed slightly larger tumors than those treated with WT Trp1 CD4^+^ cells. Tumor growth was eventually controlled in mice receiving Blimp-1-deficient Trp1 cells, though these were not fully rejected, in contrast to WT Trp1 treated mice (Figures 7J and S6H). A significant decrease in tumor control was observed in *Ifngr1*^−*/*−^ mice treated with *Prdm1*^−*/*−^Trp1 cells (Figures 7J and S6H), which is consistent with the data in Figure 1F and the idea that both helper and cytotoxic activities contribute to the potent anti-tumor activity of Trp1 cells in the context of ACTs. On average, 30% of Blimp-deficient Trp1 cells still expressed GzmB, potentially explaining the tumor control observed in 50% of *Ifngr1*^−*/*−^ recipients treated with *Prdm1*^−*/*−^Trp1 cells. We were not able to extend these experiments beyond 34 days to evaluate tumor relapse, as *Prdm1*^−*/*−^CD4^+^ T cells have been documented to promote systemic autoimmune toxicities (Martins et al., 2006). These data support Blimp-1-mediated control of the cytotoxic activity of Trp1 Th-ctx cells. Furthermore, both helper and lytic activity are vital to control tumor growth in the ACT setting.

## Discussion

Here, we found CD4^+^ T cells with polyfunctional helper and cytotoxic activity among TCR transgenic cells in models of adoptive transfer and within polyclonal CD4^+^ T cell populations in the mouse fibrosarcoma and human melanoma tumor microenvironment. These cells expressed high amounts of T-bet and IFN-γ and exhibited a Th1 phenotype but also produced the cytotoxic molecules GzmB and TNF-α. GzmB expression was independent of both T-bet and Eomes. Further analysis of the transcriptome of Trp1 Th-ctx cells revealed increased expression of *Prdm1* (Blimp-1) and several of its target genes (Bankoti et al., 2017). The transcriptome of activated CD4^+^ TILs from αCTLA-4-treated sarcoma showed a similar pattern of expression, marked by a decrease in the Tfh-cell-associated genes *Tcf7* and *Bcl6*. This transcriptional program of tumor-infiltrating CD4^+^ Th-ctx cells was similar to that shown in viral-reactive CD4^+^GzmB^+^ T cells (Donnarumma et al., 2016) but contrasts with prior studies showing T-bet-dependent differentiation of CD4^+^GzmB^+^ in a viral model (Hua et al., 2013). Blimp-1 was critical not only to the expression of GzmB by CD4^+^ T cells but also to αCTLA-4-driven anti-tumor immunity, as Blimp-1-deficient T cells were unable to control MCA205 tumor growth when mice were treated with αCTLA-4. Despite equivalent expression of T-bet and IFN-γ, both Blimp-1-deficient CD4^+^ and CD8^+^ T cells failed to produce GzmB in response to αCTLA-4, suggesting that Blimp-1 is a key factor controlling the *in vivo* differentiation of cytotoxic T cells following αCTLA-4. Indeed, transfer of Blimp-1-deficient CD4^+^ T cells into tumor-bearing-Rag-1-deficient mice did not control MCA205 tumor growth to the same extent as transfer of WT CD4^+^ T cells.

Our data also underscore the relevance and superior potency of CD4^+^ T cells bearing a polyfunctional Th cell with predominant Th1 activity and cytotoxic activity (Th-ctx). This is particularly relevant in ACT models, where loss of both perforin-1-mediated killing and sensitivity to IFN-γ resulted in significantly reduced tumor control. While complete loss of tumor control was not demonstrated in *Ifngr1*^−*/*−^ mice treated with *Prf1*^−*/*−^Trp1 cells, this may be due to previously reported Prf-1-independent, GzmB-dependent cytotoxicity (Boivin et al., 2009, Kurschus et al., 2004).

Our data highlight the central role of IL-2 in determining cell fate decisions within tumors. A deeper understanding of its impact in determining cellular phenotype and function may prove critical to optimization of current anti-cancer therapies and provide new opportunities to enhance them. IL-2 receptor signaling induces expression of Blimp-1 via STAT5, which is shown to oppose Tfh cell differentiation (DiToro et al., 2018, Johnston et al., 2009). Our findings suggest that this mechanism may orchestrate the differentiation of cytotoxic CD4^+^ T cells within tumors following treatment with Treg-cell-depleting agents. IL-2 sequestration by Treg cells restricted the differentiation of CD4^+^ Th-ctx cells *in vivo*. Our group and others previously demonstrated that maximal anti-tumor activity of αCTLA-4 antibodies depends both on blockade of the CTLA-4 co-inhibitory molecule and on intra-tumoral depletion of Treg cells (Arce Vargas et al., 2018, Peggs et al., 2009, Selby et al., 2013, Simpson et al., 2013). Because fragment crystallizable receptor (FcR) co-engagement may be important for the action of αCTLA-4 in addition to its Treg-cell-depleting activity (Waight et al., 2018), we extended our studies to include the Foxp3^DTR^ model, investigating the impact of Treg cell depletion independently of CTLA-4-blockade or FcγR co-engagement. GzmB expression was increased following Treg cell depletion and decreased following IL-2 neutralization in this model, suggesting that the increase in IL-2 amounts after Treg cell depletion is sufficient to drive differentiation of CD4^+^ Th-ctx cells.

Blimp-1 deficiency did not impact GzmB expression in adoptively transferred Trp1 cells as much as in polyclonal endogenous CD4^+^ TILs. This might be due to the higher availability of IL-2 in the adoptive T cell transfer model, resulting from Treg cell depletion and overall lymphodepletion driven by αCTLA-4 treatment and irradiation, respectively. This is in addition to higher secretion of IL-2 by activated CD4^+^ Trp1 cells, which could partially bypass Blimp-1 deficiency, as observed with the addition of exogenous IL-2 to MCA205 tumors in Blimp-1 deficient mice. IL-2 activates nuclear factor κB (NF-κB) signaling, and NF-κB binding sites have been identified in both mouse and human *GzmB* promoters (Huang et al., 2006, Zhou and Meadows, 2003). Moreover, STAT5 can directly bind to GzmB promoter region (Verdeil et al., 2006), thus potentially explaining the ability of high-dose IL-2 to bypass Blimp-1 deficiency.

Our findings suggest that Treg cells control the acquisition of cytotoxic activity by CD4^+^ T cells via competition for IL-2 availability. Furthermore, Blimp-1 and T-bet function as independent controllers of cytotoxic and helper activity in tumor-infiltrating CD4^+^ T cells, and both of these activities are critical to maximal anti-tumor activity of polyfunctional CD4^+^ Th-ctx cells. Our findings argue for the therapeutic potential of approaches focused on maximizing the impact on CD4^+^ Th-ctx activity through manipulation of the Blimp-1/Bcl6 axis in tumor-reactive CD4^+^ T cells.

## STAR★Methods

### Key Resources Table

**Table:** 

REAGENT or RESOURCE	SOURCE	IDENTIFIER
**Antibodies**		

Anti-mouse FoxP3-PE (FJL-16 s)	ThermoFisher	Cat#12-5773-82; RRID:AB_465936
Anti-mouse FoxP3-eFluor450 (FJL-16 s)	ThermoFisher	Cat#48-5773-82; RRID: AB_1518812
Anti-mouse 4-1BB-biotin (17B-5)	ThermoFisher	Cat#13-1371; RRID:AB_466603
Anti-mouse CD3-PECy.7 (145-2C11)	ThermoFisher	Cat#25-0031; RRID:AB_469571
Anti-mouse CD3-BUV737 (17A2)	BD Biosciences	Cat#564380; RRID: AB_2738781
Anti-mouse CD4-BUV496 (GK1.5)	ThermoFisher	Cat#564667; RRID:AB_2722549
Anti-mouse CD45-BUV563 (30-F11)	BD Biosciences	Cat#565710; RRID:AB_2722550
Anti-mouse CD5 (53-7.3)	ThermoFisher	Cat#45-0051; RRID:AB_914332
Anti-mouse CD8-BUV805 (53-6.7)	BD Biosciences	Cat#564920; RRID:AB_2716856
Anti-mouse CD8-BV650 (53-6.7)	ThermoFisher	Cat#100742; RRID:AB_2563056
Anti-mouse CTLA-4-BV605 (UC10-4B9)	ThermoFisher	Cat#106323; RRID:AB_2566467
Granzyme B monoclonal antibody (GB11), APC	ThermoFisher	Cat#GRB05; RRID:AB_2536539
Anti-mouse GITR-eFluor450 (DTA-1)	ThermoFisher	Cat#48-5874; RRID:AB_1944395
Anti-mouse CD25 BV510 (PC61)	BioLegend	Cat#102041; RRID: AB_2562269
Anti-mouse Lag3 –BV650 (C9B7W)	BioLegend	Cat#125227; RRID:AB_2687209
Anti-mouse CD69 – BN786 (H1.2F3)	BD Biosciences	Cat#564683; RRID:AB_2738890
Anti-mouse CD101-PE-Cy7 (Moushi101)	ThermoFisher	Cat#25-1011; RRID:AB_2573378
Anti-mouse CD44-AF700 (IM7)	ThermoFisher	Cat#56-0441-80; RRID:AB_494012
Anti-mouse IL-2 –APC (JES6-5H4)	ThermoFisher	Cat#17-7021-82; RRID:AB_469490
Anti-mouse GM-CSF-PE (MP1-22E9)	ThermoFisher	Cat# 12-7331-82; RRID:AB_466205
Anti-mouse TNFa –PE-Cy7 (MP6-XT22)	BioLegend	Cat# 506323; RRID:AB_2204356
Anti-mouse CD45- BUV563 (30-F11)	BD Biosciences	Cat# 565710; RRID: AB_2722550
Anti-mouse IFNγ-AlexaFluor488 (XMG1.2)	BioLegend	Cat#505813; RRID:AB_493312
Anti-mouse Ki67-eFluor450 (SolA15)	ThermoFisher	Cat#48-5698; RRID:AB_11151155
Anti-mouse NK1.1-eFluor450 (PK136)	ThermoFisher	Cat#48-5941; RRID:AB_2043877
Anti-NK1.1-AlexaFluor700 (PK136)	ThermoFisher	Cat#56-5941; RRID:AB_2574505
Anti-mouse PD-1-PE-Dazzle594 (29F.1A12)	BioLegend	Cat#135228; RRID:AB_2566006
Anti-mouse Prf-1 PE (S16009B)	BioLegend	Cat#135228; RRID:AB_2721640
Anti-mouse IL-2-APC (JES6-5H4)	ThermoFisher	Cat#17-7021-82; RRID: AB_469490
Anti-human CD4-AlexaFluor700 (OKT4)	ThermoFisher	Cat#56-0048; RRID:AB_914326
Anti-mouse CD38 –FITC (90)	ThermoFisher	Cat#11-0381-81; RRID:AB_465023
Anti-mouse T-bet-PE (4B10)	BioLegend	Cat# 644810; RRID: AB_2200542
Anti-human Foxp3-PE (PCH101)	ThermoFisher	Cat#12-4776; RRID:AB_1518782
Anti-CTLA-4 9H10	BioXcell	BE0131
Anti-IL-2 (JES6-1A12)	BioXcell	BE0043
Anti-IL-15 (AIO.3)	BioXcell	BE0315

**Biological Samples**		

Tissue sections of formalin-fixed and paraffin-embedded tumor samples	MSKCC	N/A

**Chemicals, Peptides, and Recombinant Proteins**		

Ionomycin	Sigma	Cat#I0634
Streptavidin-BV650	BioLegend	Cat#405232
Streptavidin-BV711	BioLegend	Cat#405241
Viability dye eFluor780	ThermoFisher	Cat#65-0856
Phorbol 12-myristate 13-acetate (PMA)	Sigma	Cat#P8139
OVA_323-339_ (ISQAVHAAHAEINEAGR) peptide	Pepscan	Custom synthesis
Trp1_106-130_ (GHNCGTCRPGWRGAACNQKILTVR) peptide	Pepscan	Custom synthesis

**Critical Commercial Assays**		

CellTrace CFSE cell proliferation kit	ThermoFisher	Cat#C34554
CellTrace Violet cell proliferation kit	ThermoFisher	Cat#C34557
Fixation/Permeabilization solution kit	BD Biosciences	Cat#554714
FoxP3/Transcription Factor Staining Buffer Set	ThermoFisher	Cat#00-5523
Liberase TL	Roche	Cat#05401020001
Recombinant DNase I	Roche	Cat#000000004716728001
Mouse GzmB ELISPOT	R&D	Cat#EL1865
Mouse CD4 Beads (L3T4)	Miltenyi	130-117-043
GranToxiLux	OncoImmunin	Cat#GTL7028

**Deposited Data**		

Microarray dataset	This study	GEO: GSE141540

**Experimental Models: Cell Lines**		

B16	ATCC	CRL-6323
B16-OVA	MSKCC	N/A
MCA205	Gift from L. Galluzzi	N/A

**Experimental Models: Organisms/Strains**		

Mice: C57BL/6	Charles River Laboratories	Cat# 027
Mice: *Tbx21*^−/−^	Gift from G. Lord (KCL)	N/A
Mice: *Prdm1*^*fl/fl*^	Gift from T. Korn (TUM)	N/A
Mice: *Cd4*^*cre*^	Gift from B.Seddon (UCL)	N/A
Mice: Trp1	Muranski et al., 2008, Quezada et al., 2010	N/A
Mice: Trp1 *Prf1*^*−/−*^	Quezada et al., 2010	N/A
Mice: *Ifngr1*^*−/−*^	Gift from G. Kassiotis (The Crick)	N/A
Mice: *Rag1*^*−/−*^	Gift from G. Kassiotis (The Crick)	N/A
Mice: OT II	Charles River	Cat# 643

**Oligonucleotides**		

qPCR primers	This study	N/A

**Recombinant DNA**		

retroviral vector pMP71	Hotblack, 2017	N/A

**Software and Algorithms**		

FlowJo 10.0.8	Tree Star	N/A
R	Cran	R-project
Prism v6 and v7	GraphPad Software	N/A
QuPath v.0.1.2	Open Source https://qupath.github.io/	N/A

### Lead Contact and Materials Availability

Inquires for further information or requests for resources and reagents should be directed and will be fulfilled by the lead contact Sergio A. Quezada (s.quezada@ucl.ac.uk). This study did not generate new unique reagents.

### Experimental Model and Subject Details

#### Mice

C57BL/6 mice were purchased from Charles River Laboratories, UK. *Tbx21* ^*−/−*^ mice were a kind gift from G. Lord (King’s College, London, UK), *Cd4*^*cre*^ from B. Seddon (UCL, UK), *Prdm1*^*fl/fl*^ mice (Shapiro-Shelef et al., 2003) from T. Korn (TUM, Munich, Germany), *Rag1*^*−/−*^ and *Ifngr1*^*−/−*^ mice from G. Kassiotis (The Crick Institute, London, UK). Trp1 mice carry the following mutation: *Rag1*^*tm1Mom*^ *x Tyrp1*^*B-w*^*x CD45.1*^*+/+*^ (Muranski et al., 2008, Quezada et al., 2010). Trp1 *Prf1*^*−/−*^ additionally carry the following mutation: *Prf1*^*tm1Sdz*^. All transgenic mice were of C57BL/6 background, bred in Charles River Laboratories (Trp1, Trp1 x *Prf1*^*−/−*^, OT-II-*CD45.1*^*+/+*^, *Tbx21*^*−/−*^) or University College London (*Prdm1*^*fl/fl*^ *Cd4*^*cre*^) animal facilities. Mice were 6 to 10 weeks old and age- and sex-matched within experiments. For adoptive transfer experiemnts with Trp1 and Trp1x*Prf1*^*−/−*^ cells C57BL/6J 5-7 weeks old male mice were used. All animal studies were performed under University College London and UK Home Office ethical approval and regulations.

#### Cell lines and tissue culture

MCA205 sarcoma tumor cells (gift from L.Galluzzi) were cultured in DMEM (Sigma) supplemented with 10% fetal bovine serum (FBS, GIBCO Sigma), 100 U/mL penicillin, 100 μg/mL streptomycin and 2 mM L-glutamine (all from GIBCO). B16-F1 (ATCC, CRL-6323) and B16-OVA cells were cultured in RPMI1640 media supplemented as above. Tumor cell lines were routinely tested and shown to be free of Mycoplasma contamination.

#### Human Samples

Presented human data derives from the phase II trial of systemic ipilimumab in combination with local melphalan for patients with in-transit melanoma (clinicaltrials.gov; *NCT01323517;* Ethics: IRB#10-101, MSKCC) included patients with unresectable stage IIIB–IV melanoma with recurrent melanoma. Patients were treated with melphalan and Ipilimumab (10 mg/kg × 4 doses, starting from days 7 to 21 after isolated limb infusion) as described (Ariyan et al., 2018). Research biopsies were taken just prior to limb infusion, after limb infusion (7–15 days), and 3 weeks after the last dose of Ipilimumab. Patients demograhpic can be found in (Ariyan et al., 2018). Tissue sections of formalin-fixed and paraffin-embedded tumor samples were provided by C.E. Ariyan (MSKCC).

### Method Details

#### Tumor experiments

6-8 weeks old C57BL/6, Foxp3^DTR^, *Tbx21*^*−/−*^*, Prdm1*^*−/−*^*Cd4*^*cre*^ *or Prdm1*^*fl/fl*^ mice were injected subcutaneously with 4 × 10^5^ MCA205 cells re-suspended in PBS. If not indicated otherwise MCA205-bearing mice were treated with 100 μg αCTLA-4 (clone 9H10, BioXcell) at days 6,9 and 12 post tumor incoculation. For treatment of *Tbx21*^*−/−*^ mice αCTLA-4 clone 4F10-IgG2a (Evitria) was used. For cytokine neutralizing αIL-2, αIL-15 or αIL-7 (200 μg) administration started at day 6 following 2 additional doses. IL-2 (Peprotech) was administered intratumorally and day 6, 8 and 10 at the dose of 4000 IU. Therapeutic antibodies: αCTLA-4 (9H10), αCD4 (GK1.5) and αCD8 (2.43), αIL-2 (JES-6-1A12), αMHC-II (M5/114), αIL-15 (AIO.3) and αIL-7 (M25) were purchased from BioXcell. Tumors were measured at least twice weekly and mice were euthanized when any orthogonal tumor diameter reached 150 mm. Tumor volume was calculated as 4/3πabc, where a, b, and c are radii.

#### Adoptive T cell transfer

C57BL/6 or *Ifgr1*^*−/−*^ mice were injected subcutaneously with 2.5 × 10^5^ B16, 2.5 × 10^5^ B16-OVA cells re-suspended in PBS. For adoptive cell transfer experiments melanoma- bearing mice were treated or not with 5 Gy of body irradiation followed by adoptive transfer of transgenic T cells. Trp1, Trp1x*Prf1*^*−/−*^ or OT-II cells were isolated from spleen and LN of naive Trp1, Trp1x*Prf1*^*−/−*^ or OT-II mice on the day of adoptive transfer, respectively and purified with CD4^+^ beads (130-117-043, Miltenyi) according to the manufacturer’s protocol. 0.6 × 10^5^ Trp1; 0.6 × 10^5^ Trp1x*Prf1*^*−/−*^ *or* 3 × 10^5^ OT-II cells were re-suspended in PBS administered intravenously. Mice were tereated or not with 200 μg αCTLA-4 i.p on day 8 and 100 μg αCTLA-4 on days 11 and 14. Cytokine neutralizing antibodies were administered at the time points indicated in Figures S1, S2, and S3. Mice in Th conditions received 10^6^ of irradiated (150 Gy) GVAX cells (GM-CSF-expressing B16 cells).

*Rag1*^*−/−*^ mice were injected subcutaneously with 3 × 10^5^ MCA205 cells re-suspended in PBS. Mice received polyclonal Blimp-1-sufficient or Blimp-1-deficient CD4^+^ T cells. Polyclonal CD4^+^ TILs and dLN cells were isolated from TILs and dLN of αCTLA-4-treated mice at day 11 post tumor inoculation and purified with CD4+ beads (130-117-043, Miltenyi) and incubated with αCD8 (50 μg) for 30 minutes at for CD8^+^ T cell depletion. Tumor-bearing *Rag1*^*−/−*^ recipients were not irradiated. For cytokine neutralizing in adoptive transfer setting αIL-2 or αIL-7 (200 μg) administration started at day 11 following 2 additional doses. Tumors were measured at least three times weekly and mice were euthanized when any orthogonal tumor diameter reached 150 mm. Tumor volume was calculated as 4/3πabc, where a, b, and c are radii.

#### Adoptive transfer of Trp1 TCR transduced cells

The transduction procedure was performed according to (Hotblack et al., 2018). Briefly, the TRP1 TCR (Kerkar et al., 2011, Muranski et al., 2008) was cloned into the retroviral vector pMP71 with a 2A sequence separating the Vα3.2 and Vβ14 chains, followed by an internal ribosome entry site (IRES) truncated CD19 sequence. The TCR was codon optimized and also contains an extra cysteine residue in the constant chains to enhance pairing of the α and β chains (Hotblack, 2017). To generate TRP1 retroviral particles, Phoenix-Eco (PhEco)-adherent packaging cells (Nolan Laboratory) were transiently transfected with retroviral vectors for the generation of supernatant containing the recombinant retrovirus required for infection of target cells, as described previously. The PhEco-adherent packaging cells were transfected using Genejuice (Merck) with the pCL-eco construct and the TRP1 TCR vector according to the manufacturers’ instructions. *Prdm1*^*fl/fl*^ or *Prdm1*^−/−^*Cd4*^*cre*^ CD4^+^ T cells were purified by magnetic selection according to the manufacturer’s instructions (Miltenyi). Sorted cells were activated with concanavalin (Con) A (2 μg/mL) and IL-7 (1 ng/mL) for 24 h, and then 2 × 10^6^ activated T cells were incubated for a further 72 h with retroviral particles on retronectin-coated (Takara-Bio) 24-well plates, in the presence of IL-2 (100 U/mL; Roche). Cells were stained with anti-TCR Vb14 antibody to confirm transduction efficiency. Transduced cells were administerd intravenously into melanoma-bearing mice 72 h after transduction. The receipient mice were treated with therapeutic antibodies at the time points indicated at Figure S6E. Tumors were measured at least three times weekly and mice were euthanized when any orthogonal tumor diameter reached 150 mm. Tumor volume was calculated as 4/3πabc, where a, b, and c are radii.

#### Mouse tissue processing

Mice used for functional experiments were sacrificed on day 13 (MCA205) or 17/18 (melanoma) after tumor implantation, and LN cells and tumor-infiltrating lymphocytes were isolated as previously described (Quezada et al., 2006, Simpson et al., 2013). Briefly, lymph nodes and tumors were dissected into RPMI medium. Lymph nodes were dispersed through a 70-μm filter whereas tumors were mechanically disrupted using scissors, digested with a mixture of 0.33 mg/ml DNase (Sigma-Aldrich) and 0.27 mg/ml Liberase TL (Roche) in serum-free RPMI for 30 min at 37°C, and dispersed through a 70-μm filter. Cells were either re-suspended in FACS buffer (PBS with 2% FBS and 2 mM EDTA), re-stimulated with cognate antigen, stimulated with PMA and Ionomycin or further cell type-specific purification was performed. Tumor-infiltrating CD4^+^ T cells were purified using CD4 positive selection (FlowComp; Invitrogen) according to the manufacturer’s instructions. Purified CD4^+^ T cells from tumors or bulk cells from LNs were restimulated for 4 h at 37°C with 5 × 10^4^ DCs and 1μM of Trp1 or OVA peptide followed by addition of brefeldin A (BD) for 2 more hours. Polyclonal CD4^+^ T cells were restimulated with phorbol 12-myristate 13-acetate (PMA, 20 ng/mL) and ionomycin (500 ng/mL; Sigma Aldrich) for 4 hours at 37°C in the presence of GolgiPlug (BD Biosciences).

#### Flow cytometry

Directly conjugated antibodies employed for flow cytometry are listed in Key Resourse Table. Surface staining was performed at 4°C with antibodies re-suspended in PBS with 2% FBS and 2 mM EDTA. Staining of FoxP3, Ki67 and GzmB was performed using the FoxP3 Transcription Factor Staining Buffer Set (ThermoFisher). Cytokine staining was performed using Cytofix/Cytoperm buffer set (BD Biosciences). For quantification of absolute number of cells, a defined number of fluorescent beads (Cell Sorting Set-up Beads for UV Lasers, ThermoFisher) was added to each sample before acquisition and used as a counting reference. Cells were acquired using BD LSR Fortessa ob BD FACSymphony instruments.

#### Phospho-flow cytometry

For pSTAT5 staining TILs and LN cells were rested for 2h in FCS-free RPMI media followed by 10 min stimulation with 50 IU/ml of IL-2 (Peprotech) at 37°C and fixed for 30 min with Fixation/Permeabilization buffer (ThermoFisher) and Perm Buffer III (BD Phosflow) on ice followed by the intracellular staining with anti-pSTAT5 and and anti-Foxp3 antibodies. Cell were stained for 20 minutes prior to IL-2 stimulation with BD Horizon Fixable Viability Stain 450 (562247, BD Bioscience).

#### Mouse T cells activation assays

Purified CD4^+^ T cells (CD4 T cells beads, Miltenyi or CD4 Dyna Beads, Invitrogen) were labeled with CFSE or CellTrace Volet (CTV) (Thermofisher) according to manufacturer’s protocol and cultured in RPMI 1640 complete medium supplemented with 10% fetal bovine serum (FBS, GIBCO Sigma), 100 U/mL penicillin, 100 μg/mL streptomycin and 2 mM L-glutamine (all from GIBCO) together with DC and irradiated feeder cells (40 Gy) in 2:1:1 ratio for 72 or 96h. Polyclonal CD4^+^ T cells were stimulated with αCD3 (2C11) and αCD28 (37.51) (BioXcell); OT-II cells with OVA_323-339_ (ISQAVHAAHAEINEAGR) peptide (Pepscan) and Trp1 cells with Trp1_106-130_ (SGHNCGTCRPGWRGAACNQKILTVR) peptide (Pepscan) at concentration indicated in the Figure 2. Cells were additionally supplemented with IL-2, IL-15 or IL-7 (Peprotech) at a concentration indicated in the Figure 2. Mouse CD4^+^ T cells were cultured with αCD25 (PC61, BioXcell) and αIL-2 (JES6-1A12, BioXcell), added to the culture 24h post stimulation with αCD3 and αCD28 in a concentration of 5 μg/ml.

#### Human T cells activation assays

Human PBMCs were isolated from healthy volunteers’ blood (Ethics: UCL REC Project ID 8261/001). FACS-purified human CD4^+^ T cells were cultured in RPMI complete medium with irradiated autologous feeders cells in 1:1 ratio for 96h, stimulated with αCD3 (OKT3) and αCD28 (9.3;BioXcell). To some wells αCD25 antibody (Basiliximab; Novartis) was added 48 hours post αCD3 + αCD28 stimulation. For Treg suppression assays FACS-purified naive human CD4^+^ T cells were co-cultured with autologous Treg cells at indicated ratios (Figure 2), to some wells IL-2 (Peprotech) was added.

#### Cytotoxicity assays

For *in vitro* killing assays Th-ctx Trp1 cells were purified from tumors and draining LN at day 7 post transfer and expanded for 72h with Trp1 peptide (1 μM), DCs and 20 IU IL-2. Target B16 cells were preconditioned with IFNγ overnight to increase MHC-II expression and labeled with 5 μM CFSE (ThermoFisher) and plated together with control cell line labeled with 0.5 μM CFSE 1:1 on 96-well plate. Effector Trp1 cells were labeled with CTV (ThermoFisher) and co-culture with target cells for 16 hours. Cells were stained with live/dead dye (Viability dye eFluor780) for FACS analysis. OT-II cells were activated with DCs and OVA peptide (1 μM) and either plated with B16-OVA and control cell line for 16h or cytotoxicity was measured using the GranToxiLux-PLUS kit (Oncoimmunin, Gaithersburg, MD, USA), according to the manufacturer’s instructions. Briefly, target cells were identified by labeling with a target fluorescent probe (TFL-4) and with a nuclear fluorescent labeling probe (NFL1), to exclude cells that had died before the start of the assay. Effector (OT-II) and target cells were mixed at a ratio of 5:1 and co-incubated in the presence of a FITC-conjugated GzmB substrate for 2 hours. Cytotoxic activity was detected by the cleavage of the substrate, which released FITC and thus rendered target cells fluorescent.

#### Granzyme B ELISPOT

Purified CD4^+^ TILs (800 cells/well) and LN cells (10.000 cells/well) form MCA205 αCTLA-4 treated tumors were cultured on anti-GzmB coated ELISPOT Plate (R&D) for 24h with unpulsed DC or MCA205-pulsed DCs and 50 μg/ml anti-MHC-II (M5/114). ELISPOT Assay was performed according to manufacturer’s protocol.

#### Immunohistochemistry

The following primary antibodies were used for the multiplex immunohistochemistry (IHC): anti-FoxP3 (clone 236A/E7; dilution 1:100, a gift from Dr. G. Roncador, CNIO, Madrid, Spain); anti-CD4 (clone 4B12; dilution 1:30, Leica Microsystems, Newcastle-upon-Tyne, UK) and GzmB (clone 11F1; dilution 1:40, Leica Microsystems, Newcastle-upon-Tyne, UK). To establish optimal staining conditions (i.e., antibody dilution and incubation time, antigen retrieval protocols, suitable chromogen) each antibody was tested and optimized on 2-4 um cut tissue sections of human reactive tonsil by conventional single immunohistochemistry using the automated platforms BenchMark Ultra (Ventana/Roche) and the Bond-III Autostainer (Leica Microsystems) according to the protocols described elsewhere (Akarca et al., 2013, Marafioti et al., 2008) Tissue sections (4um) from formalin-fixed and paraffin-embedded blocks of human tumor samples (clinincaltrials.gov: NCT01323517) were de-waxed and re-hydrated before subjected to multiplex-IHC. The procedure was performed following the principles of previously established protocols (Marafioti et al., 2003)(Marafioti et al., 2008) adapted using the Ventana Benchmark Ultra immunostainer. Briefly, tissue sections were subjected to antigen retrieval following the conventional protocol of the Ventana Benchmark Ultra and then incubated with each primary antibody for 30 minutes. Sites of labeling were detected using the peroxidase-based detection reagent conjugate (OptiView DAB IHC Detection Kit) followed by the alkaline phosphatase detection kit (UltraView Universal Alkaline Phosphatase Red Detection Kit), both from Ventana Medical Systems, Inc. After staining, samples were washed in buffers and distilled water and mounted in Apathys mounting medium (TCS Biosciences Ltb). Specificity of the staining was assessed by a hematopathologist (TM) with expertise in multiplex-immunostaining. For evaluation of protein co-expression in the cytoplasm or cell membrane, change of the single color of the chromogen was noted i.e., brown and blue gave rise to almost black labeling while co-expression of cytoplasmic and/or membranous with nuclear proteins was revealed by either brown and/or blue with the nuclear green labeling. No nuclear counterstaining with Haematoxylin was performed. Double positive CD4^+^GzmB^+^ and CD4^+^Foxp3^+^ cells were quantified in QuPath software. Briefly on average six representative areas (0.25 mm^2^) were selected within each tumor areas ranging from minimum of 2 to maximum of 10 areas. Areas with necrotic tissue were excluded from the analysis. The average cells count per 0.5 mm^2^ tumor tissue was calculated.

#### Quantitative qPCR

RNA from FACS-purified CD4^+^CD25^lo^ TILs from MCA205 tumor was extracted with RNeasy micro kit (QIAGEN) according to manufacturer’s protocol. Amount of RNA was quantified with Qubit (ThermoFisher). Synthesis of cDNA was carried out with SuperScript III reverse transcriptase (ThermoFisher). Purified cDNA was then used as template for the quantitation of the indicated genes using gene-specific primers (Table S3). qPCR was performed with QuantiTect Sybr Green PCR kit Syber reagents (QIAGEN). Values were normalized and plotted according to the expression of *Hprt1* in the same samples, using a ΔC_T_ method.

#### Th-ctx and Th Trp1 transcriptome analysis

B16-bering mice were treated with 0.6 × 10^5^ naive Foxp3^GFP^ Trp1 cells, irradiated GVAX and αCTLA-4 (Th condition) or irradiation (5Gy) and αCTLA-4 (Th-ctx condition), details Figure S1. Control mice received naive Trp1 cells only (control). 8 days after transfer Trp1 GFP^-^ (Foxp3-negative) cells were FACS purified. RNA was isolated using TRIzol (Invitrogen) according to the manufacture’s protocol. The GeneChip® Mouse Genome 430 2.0 Array (Affymetrix) was used to analyze the transcriptome. Raw expression values were normalized using the robust multi-array average (RMA) procedure (Irizarry et al., 2003) implemented in the package affy (Bioconductor). Differential gene expression analysis was carried out on all genes, or a selection of previously described transcription factors (Gerstberger et al., 2014) in the package limma (Smyth, 2004). Gene set enrichment analysis (GSEA) was conducted using the package fgsea with 1000 permutations (Sergushichev, 2016), with reactome and MSigDB C7 signature sets (Godec et al., 2016). Correction for multiple testing was carried out using the Benjamini-Hochberg method. All microarray analyses were done in the R statistical programming environment.

#### Software

Flow cytometry data were analyzed with FlowJo v10.0.8 (Tree Star). IHC data were analyzed with QuPath (v0.1.2). Statistical analyses were done with Prism (v6 and v7) (GraphPad Software). All microarray analyses were done in the R statistical programming environment.

### Quantification and Statistical Analysis

Statistical analyses were done with Prism v6 and v7 (GraphPad Software); p values were calculated using one or two-way ANOVA with Tukey post-tests (ns = p > 0.05,^∗^ p < 0.05, ^∗∗^p < 0.01, ^∗∗∗^p < 0.001, ^∗∗∗∗^p < 0.0001 for 1 way ANOVA and ^∗^p < 0.033, ^∗∗^p < 0.0021, ^∗∗∗^p < 0.0002, ^∗∗∗∗^p < 0.0001 for 2-way ANOVA). Kaplan-Meier curves were analyzed with the log-rank test. N in animal experiments refers to number of animals per experimental group.

### Data and Code Availability

The accession number for the Trp1 microarray reported in this paper is GEO: GSE141540.

## Supplemental Information

Document S1. Figures S1–S6

Table S1. Genes Differentially Expressed between Trp1 Th Cells and Trp1 Th-ctx Cells, Related to Figure 1

Table S2. Genes Encoding Transcription Factors Differentially Expressed in Trp1 Th Cells and Trp1 Th-ctx Cells, Related to Figure 1

Table S3. Oligonucleotide Sequences, Related to STAR Methods

Document S2. Article plus Supplemental Information
