# Supplementary material for: Regulatory T Cells Restrain Interleukin-2- and Blimp-1-Dependent Acquisition of Cytotoxic Function by CD4+ T Cells
Source: Immunity. 2020 Jan 14;52(1):151–166.e6. doi: 10.1016/j.immuni.2019.12.007 (PMC7369640; doi:10.1016/j.immuni.2019.12.007)
Supplement: Document S1. Figures S1–S6 [file mmc1.pdf]

Immunity, Volume *52*

Supplemental Information

Regulatory T Cells Restrain Interleukin-2- and Blimp-1-Dependent Acquisition of Cytotoxic Function by CD4$^+$ T Cells

Anna Śledzińska, Maria Vila de Mucha, Katharina Bergerhoff, Alastair Hotblack, Dafne Franz Demane, Ehsan Ghorani, Ayse U. Akarca, Maria A.V. Marzolini, Isabelle Solomon, Frederick Arce Vargas, Martin Pule, Masahiro Ono, Benedict Seddon, George Kassiotis, Charlotte E. Ariyan, Thomas Korn, Teresa Marafioti, Graham M. Lord, Hans Stauss, Richard G. Jenner, Karl S. Peggs, and Sergio A. Quezada

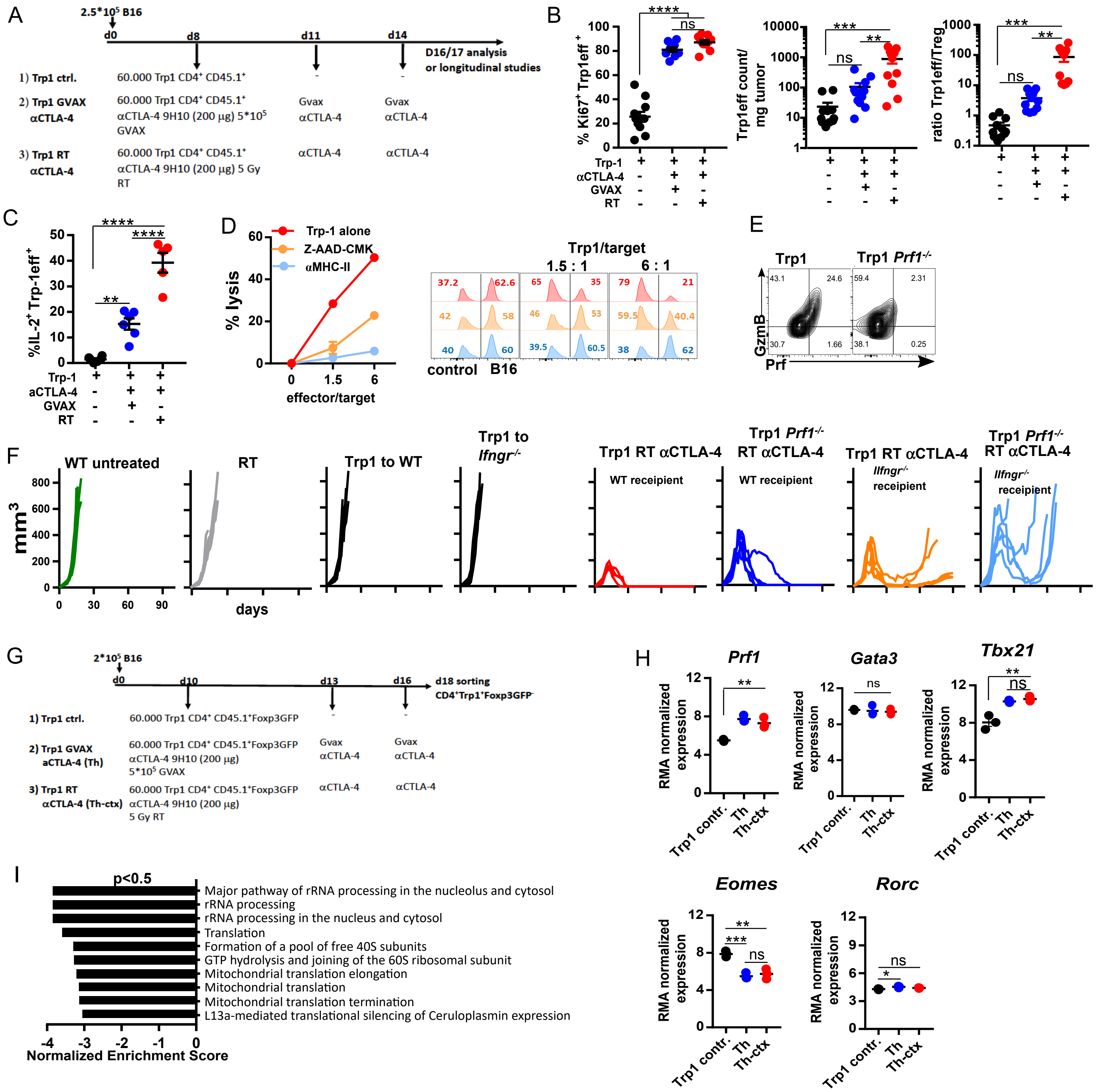

Figure S1 related to Figure 1. Tumor-reactive CD4$^+$ T cell acquire cytotoxic phenotype in tumor microenvironment following lymphopenia induced expansion.

(**A-C**) B16 tumor-bearing mice were treated at day 8 with $0.6*10^5$ Trp-1 cells alone or combine with αCTLA-4, radiation or GVAX. (**A**) Experimental schema Figure 1A. (**B**) TILs were isolated at day 16 post tumor inoculation. Quantification of Ki67-expressing Trp1 cells, number or Trp1 effector cells and Trp1 effector to all Treg ratio in tumor (n=10-11 mice/group, cumulative data of 2 independent experiments). (**C**) Quantification of IL-2-expressing cells within Trp1 compartment (N=5-6 mice/group). (**D**) In vitro killing assay. CD4$^+$Trp1$^+$ T cells were primed *in vivo*, expanded *in vitro* for 72h and co-cultured with CFSE -labelled (5 µM) B16 melanoma and CFSE-labelled (0.5 µM) control cell line with additional MHC-II blocking or in the presence of 25 µM Z-AAD-CMK for 16h. Analysis by FACS. Shown are percentage of specific lysis of B16 cells and representative FACS plots showing frequency of live B16 cells in relation to control cell line in indicated conditions. Representative data of 2 independent experiments. (**E**) Trp1 and Trp1x Prf-1-deficient cells were stimulated with Trp1 peptide (1 µM) for 72 h. Representative plots showing expression of GzmB and Prf1. (**F**) Experiment as in Figure 1E. Tumor growth curves of individual mice. (**G-I**) Transcriptome analysis (**G**) Experimental schema Figure 1D-F. (**H**) RMA normalized expression of *Prf1* and selected transcription factors in Trp1 Th and Trp1 Th-ctx condition in comparison to Trp1 control cells (**I**) Reactome pathways enrichment analysis. Shown are the highest upregulated pathway in Th condition (NES >2, $p < 0.5$).

All quantification plots: mean ± SEM, 1-way ANOVA (* $p<0.05$, ** $p<0.01$, *** $p<0.001$, **** $p<0.0001$)

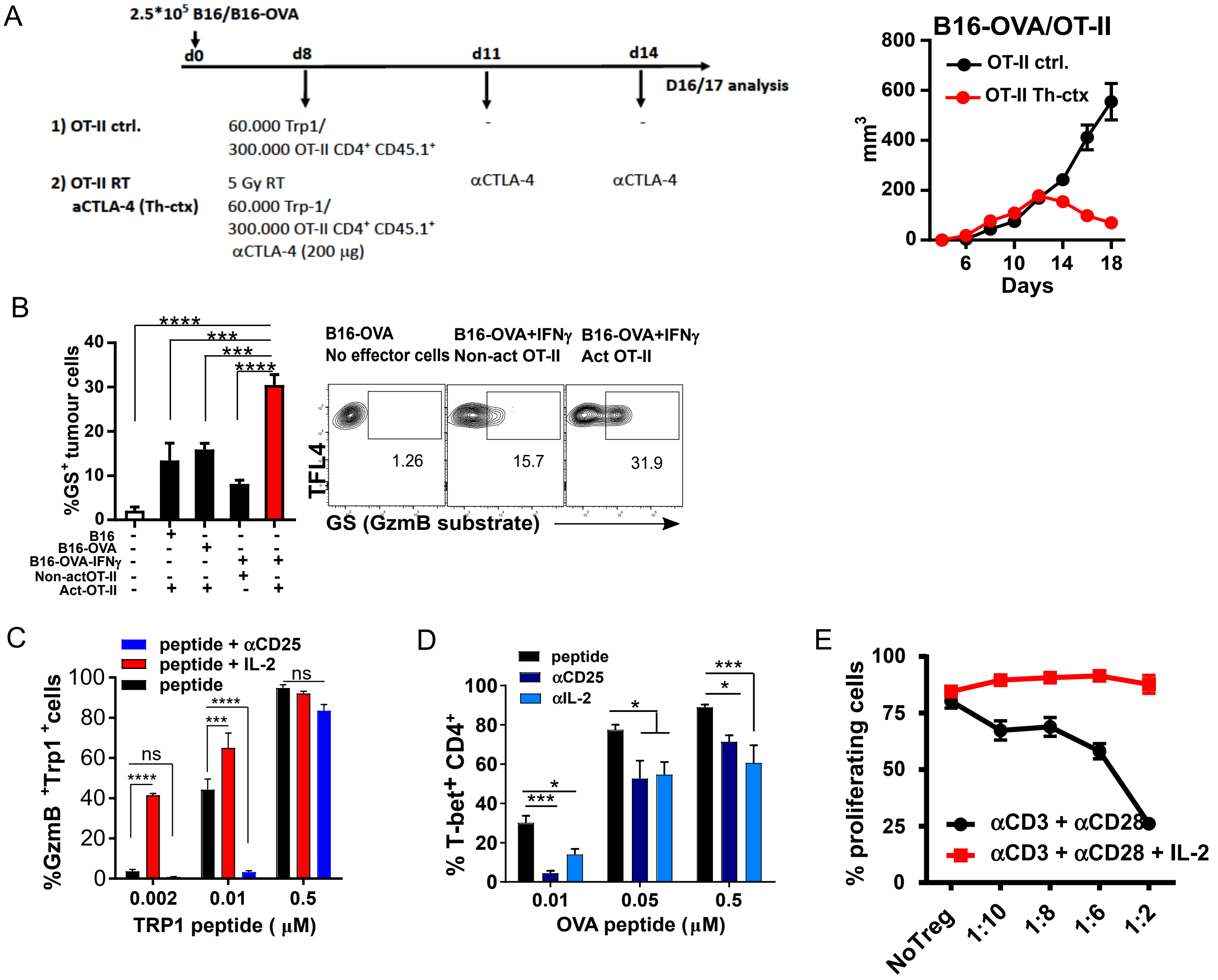

Figure S2 related to Figure 2. Blocking of IL-2 signaling in vitro decreases Gzm B expression by mouse and human CD4⁺ T cells.

(**A**) OT-II T cells transfer to B16-OVA-bearing mice. Experimental schema Figure 1G and tumor growth. (**B**) Activated or non-activated OT-II cells were culture with B16-OVA or B16 cell for 2 h in the presence of GzmB substrate. Representative plots showing GzmB substrate- positive B16 or B16-OVA cells and quantification. Representative data of two independent experiments. (**C**) CTV-labelled Trp1 cells were cultured for 72h with APC and indicated amount of peptide either with 100 IU/ml IL-2 or 5 μg/ml CD25. Quantification of GzmB-expressing cells within proliferating compartments. (**D**) CTV-labelled OT-II cells cultures as in Figure 2D with or without 5 μg/ml of indicated antibodies. Quantification of T-bet expressing CD4⁺ T cells within proliferating compartments. (**E**) Experiment as in Figure 2J. Quantification of proliferating CD4⁺ T cells in indicated conditions without and with addition of IL-2.

All quantification plots: mean ± SEM, 1-way ANOVA (* $p<0.05$, **$p<0.01$, ***$p<0.001$, ****$p<0.0001$)

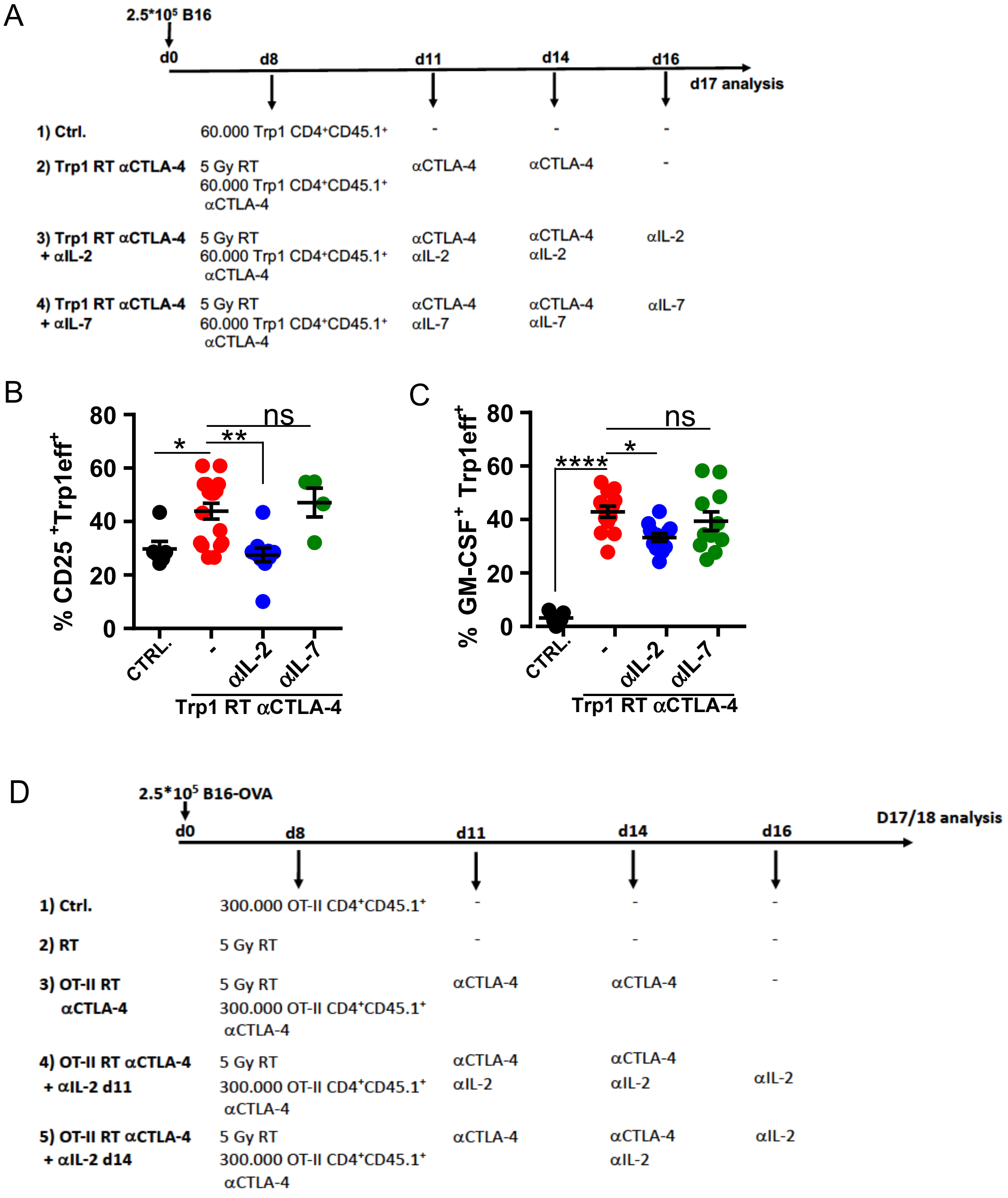

Figure S3 related to Figure 3. CD4 TILs in Th-ctx condition reduce Gzm B expression but retain Th1 phenotype upon IL-2 neutralization.

(A-C) B16 tumor-bearing mice received Trp1 cells at day 8 post tumor inoculation either after irradiation (Th-ctx) or without (Ctrl) and followed by αCTLA-4 and cytokine neutralizing antibodies treatment. **(A)** Schema of experiment in Figure 3 **(A-C)**. **(B)** Quantification of CD25- expressing cells **(C)** Quantification of GM-CSF-expressing cells within Trp1eff compartment (N= 7-13 mice/group, cumulative data of 2 independent experiments). **(D)** B16-OVA-tumour bearing mice received OT-II cells at day 8 post tumor inoculation alone or in combination with RT and cytokine neutralizing antibodies. Schema of experiment in Figure 3D-E.

All quantification plots: mean ± SEM, 1-way ANOVA (* p<0.05, **p<0.01, ***p<0.001, ****p<0.0001)

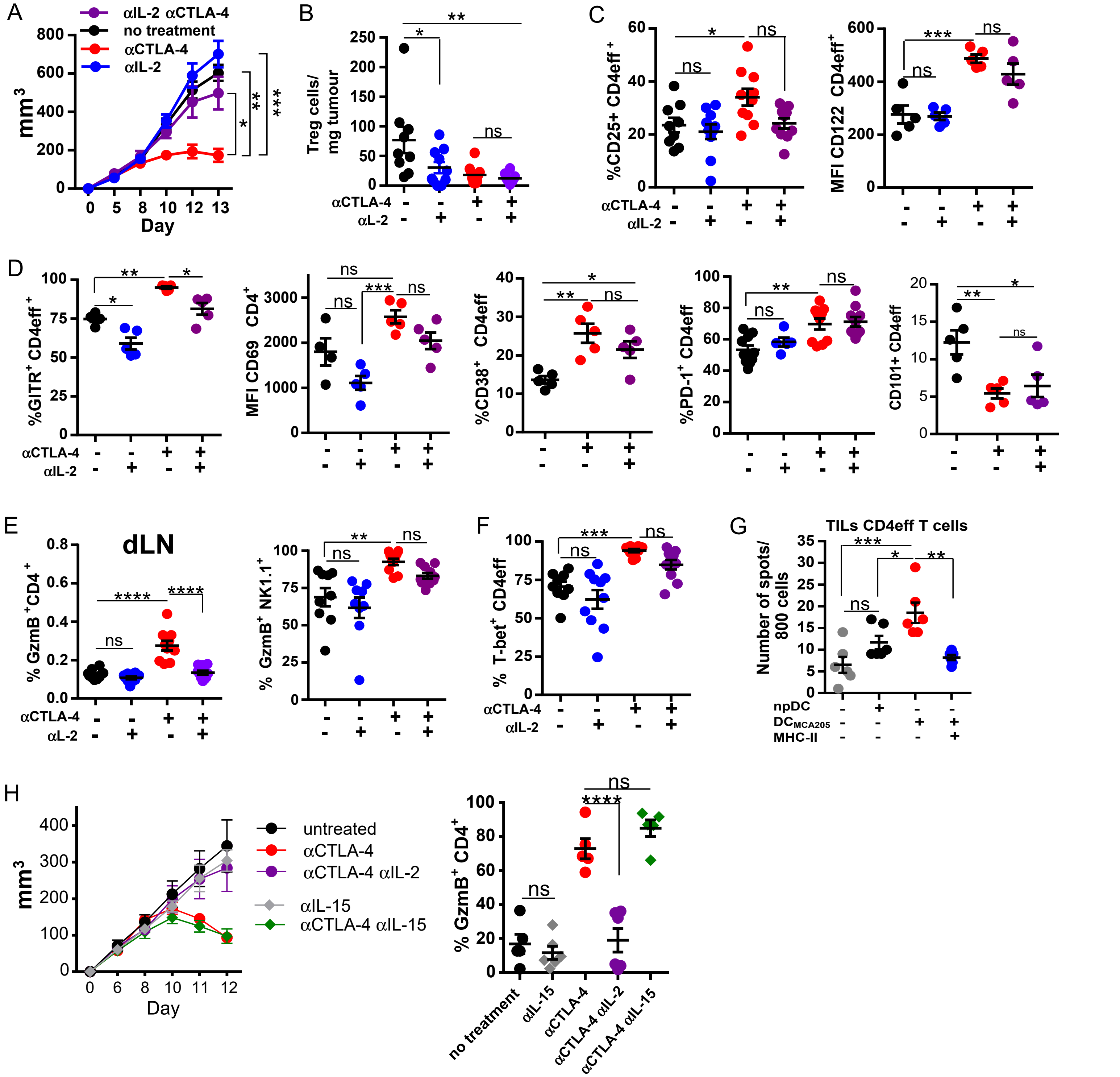

Figure S4 related to Figure 4. Increased level of available IL-2 after Treg depletion contributes to shaping T helper cells phenotype in the tumor microenvironment.

(A–F) Experiment as in **Figure 4C-G**. **(A)** Tumor growth. **(B)** Quantification of Treg cell within CD4 $^{+}$ TILs compartment at day 13 post tumor inoculation in indicated conditions as in **Figure 4C** (N=10 mice/group, cumulative data of 2 independent experiments). **(C)** Quantification of CD25 and CD122-expresing cells within CD4eff compartment (N= 5-10 mice/group, cumulative data of 2 independent experiments). **(D)** MCA205 TILs: Quantification of indicated markers within CD4eff compartment as in **Figure 4E**. **(E)** Quantification of GzmB-expressing cells within dLN Treg and NK1.1 cells compartment (N=10 mice/group, cumulative data of 2 independent experiments). **(F)** Quantification of T-bet-expressing cells within CD4+ T cells. **(G)** 800 CD4 TILs cells were cultured unstimulated or stimulated with non-pulsed DCs or MCA205-pulsed DCs on aGzmB-coated ELISPOT plate for 24h. Numbers represents GzmB spots per 800 responding CD4 T cells. **(H)** MCA205 tumor-bearing mice were treated with 100 µg of aCTLA-4 9H10 antibody on days 6, 8, and 10 after tumor implantation alone or combined with 200 µg of aIL-2 or aIL-15 on days 6, 8, 10 after tumor implantation. Growth curves of MCA205 tumors, showing the product of three orthogonal tumor diameters and quantification of GzmB expression by CD4⁺ TILs are shown (N=5 mice/group).

All quantification plots: mean ± SEM, 1-way ANOVA (* p<0.05, **p<0.01, ***p<0.001, ****p<0.0001)

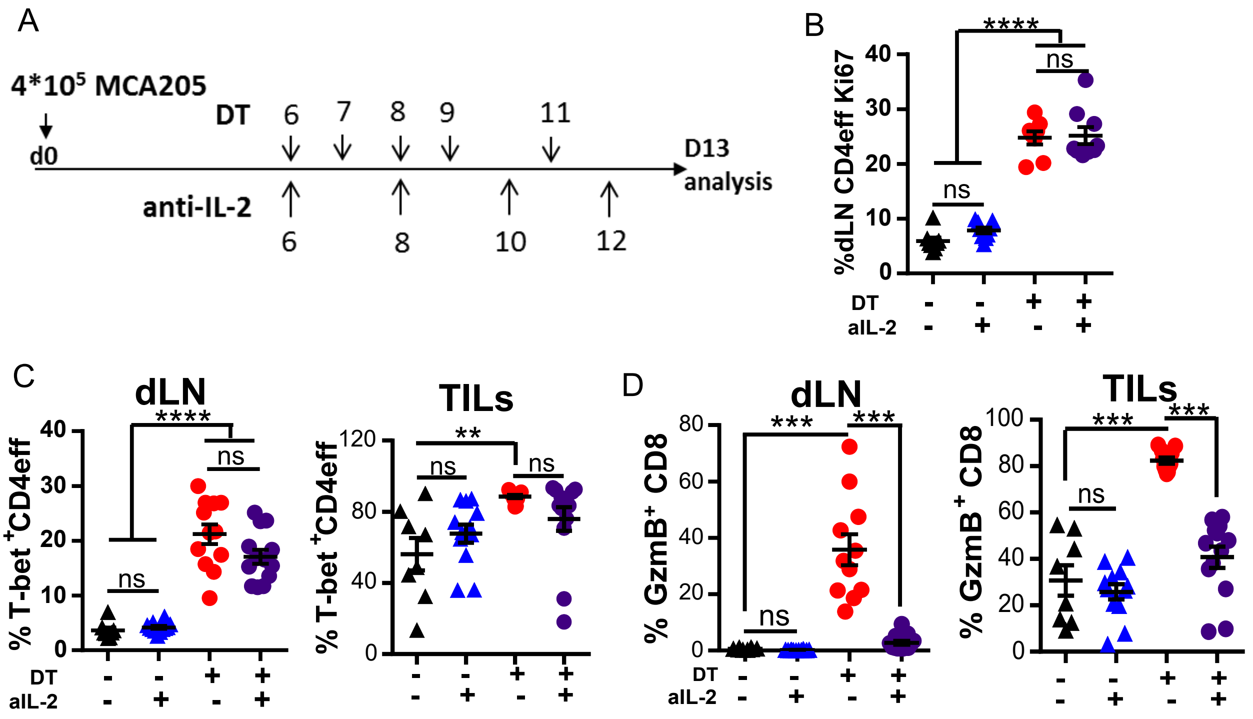

Figure S5 related to Figure 5. Treg depletion without CTLA-4 blocking is sufficient to drive GzmB expression by CD4$^+$ T cells.

(**A-D**) Foxp^{DTR} MCA205 tumor-bearing mice were treated from d6 with DT at days 6, 7, 8, 9 and 10 and aIL-2 (200µg) at days 6, 8, 10 and 12. (**A**) Schema of experiment as in Figure 5A-E. (**B**) Quantification of Ki67- expressing CD4$^+$ T cells in dLN and TILs. (**C**) T-bet-expressing CD4$^+$ T cells in dLN and TILs. (**D**) Quantification of GzmB-expressing CD8$^+$ T cells in dLN and TILs (N=10 mice/group, cumulative data of 2 independent experiments).

All quantification plots: mean ± SEM, 1-way ANOVA (* p<0.05, **p<0.01, ***p<0.001, ****p<0.0001)

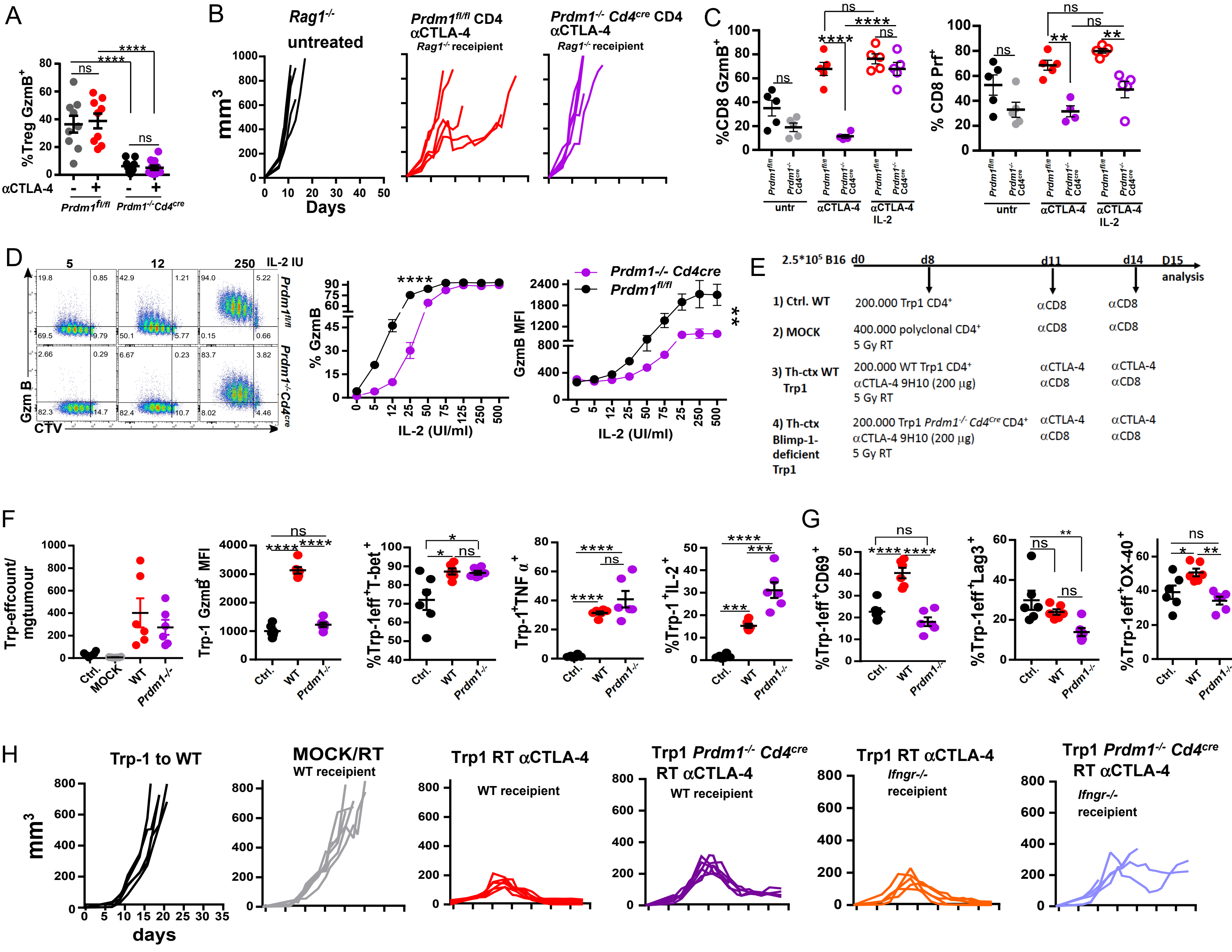

Figure S6 related to Figure 7. IL-2 milieu in tumor controls differentiation of cytotoxic CD4+ T cells in Blimp-1-dependent manner.

(A) Experiment as in **Figure 7B-C**. Quantification of Gzm B -expressing cells within Treg TILs compartment. **(B)** Experiment as in **Figure 7D.** Tumor growth of individual mice. **(C)** Experiment as in **Figure 7F.** Representative plots and quantification of GzmB-and Prf-1-expressing cells within CD8 TILs (N=5 mice/group). **(D)** CTV-labelled MACS-sorted CD4$^+$ T cells from WT and *Prdm1$^{-/-}$Cd4^{cre}* mice were cultured for 72h in the presence of APCs with 0.1 µg/ml αCD3 antibody and indicated amount of IL-2. Representative plots and quantification of GzmB expressing CD4$^+$ T cells within proliferating cell, MFI of GzmB expressing CD4$^+$ T cells within proliferating cells (n =3, representative data of 2 independent experiments). **(E-G)** Experiment as in **Figure 7G-H**. **(E)** Schema of the experiment. **(F)** Quantification of GzmB and cytokines within Trp-1 compartment in indicated conditions. **(G)** Quantification of indicated molecules within Trp-1 compartment in indicated conditions. **(H)** Experiment as in **Figure 7J**. Tumor growth curves of individual mice are shown.

All quantification plots: mean ± SEM, 1-way ANOVA (* p<0.05, **p<0.01, ***p<0.001, ****p<0.0001)
